# Endothelial Cell Self-fusion during Vascular Pruning

**DOI:** 10.1371/journal.pbio.1002126

**Published:** 2015-04-17

**Authors:** Anna Lenard, Stephan Daetwyler, Charles Betz, Elin Ellertsdottir, Heinz-Georg Belting, Jan Huisken, Markus Affolter

**Affiliations:** 1 Biozentrum der Universität Basel, Basel, Switzerland; 2 Max Planck Institute of Molecular Cell Biology and Genetics, Dresden, Germany; Duke University Medical Center, UNITED STATES

## Abstract

During embryonic development, vascular networks remodel to meet the increasing demand of growing tissues for oxygen and nutrients. This is achieved by the pruning of redundant blood vessel segments, which then allows more efficient blood flow patterns. Because of the lack of an in vivo system suitable for high-resolution live imaging, the dynamics of the pruning process have not been described in detail. Here, we present the subintestinal vein (SIV) plexus of the zebrafish embryo as a novel model to study pruning at the cellular level. We show that blood vessel regression is a coordinated process of cell rearrangements involving lumen collapse and cell–cell contact resolution. Interestingly, the cellular rearrangements during pruning resemble endothelial cell behavior during vessel fusion in a reversed order. In pruning segments, endothelial cells first migrate toward opposing sides where they join the parental vascular branches, thus remodeling the multicellular segment into a unicellular connection. Often, the lumen is maintained throughout this process, and transient unicellular tubes form through cell self-fusion. In a second step, the unicellular connection is resolved unilaterally, and the pruning cell rejoins the opposing branch. Thus, we show for the first time that various cellular activities are coordinated to achieve blood vessel pruning and define two different morphogenetic pathways, which are selected by the flow environment.

## Abstract 2

In vivo live imaging of zebrafish embryos reveals the diverse cellular activities involved in the pruning of unwanted blood vessels, including the self-fusion of endothelial cells.

## Author Summary

The blood vasculature circulates gas, nutrients, hormones, and metabolites to all organs of the body. It is indispensable for survival and already functions at very early stages of embryonic development. At this point, new blood vessels form mainly through angiogenesis—the outgrowth of new vessels from existing ones. New vascular sprouts connect to each other to form functional loops with blood flow, a process termed anastomosis. Vascular plexuses formed in this way subsequently remodel to a final structure with efficient flow patterns. Remodeling often involves pruning or regression of unnecessary branches, leading to a simplification of the network. Our in vivo live imaging studies of the pruning process in the zebrafish embryo show that vessel regression occurs through cell rearrangements, wherein cells consecutively migrate out of the pruning branch. As a result, the initially multicellular vessel is reduced to a single cell connection that is eventually resolved when the last cell incorporates into the neighboring branch. If the lumen is maintained in the pruning vessel, the process involves transient formation of a unicellular tube through cell self-fusion. Thus, we show how a variety of cellular activities are coordinated to achieve vessel pruning.

## Introduction

The vascular system of vertebrates distributes oxygen, nutrients, metabolites, and blood cells to and from all organs of the body. A complex network of interconnected vascular tubes develops and already functions at early stages of embryonic development and continues to expand and to remodel as the animal grows [1]. Blood vessels invade avascular tissue areas by sprouting angiogenesis, which is defined by the branching of new vessels from existing ones [2,3]. Later on, angiogenic sprouts connect to each other in a process termed vascular anastomosis [4,5].

Emerging vascular beds are often organized in a plexus, which, in a primitive state, constitutes a nonhierarchical network of blood vessels. As the demand for oxygen and nutrients during organ or embryonic growth increases, these primitive networks become remodeled to allow more efficient blood transport. Such vascular remodeling involves changes in vessel diameter as well as pruning of supernumerary vascular branches [4]. The pruning process has been studied in the vasculature of the mouse retina [6,7] and extra-embryonic vessels of chick and mouse embryos [8–10] and, more recently, in several vascular beds of the zebrafish embryo [11,12]. Taking advantage of the ease of performing live imaging in the zebrafish embryo, the latter studies showed that regression of vessels occurs through endothelial cell rearrangements typically in the absence of apoptosis. Importantly, these studies showed that pruning is regulated by hemodynamic forces, since blood vessel regression was preceded by changes in blood flow patterns due to rewiring of the vascular plexus.

We have previously shown that blood vessel fusion occurs in discrete morphogenetic steps, in which cell rearrangements play a crucial role in the transformation of blood vessel architecture from a unicellular to multicellular configuration [13,14]. The studies by Kochhan et al. [12] pointed out that cell activities involved in vessel pruning might resemble those during vessel fusion but in reversed order. However, the anatomical localization of the vessels analyzed in that report made it impossible to describe the pruning process at high cellular resolution. To analyze the pruning process in more detail, we have now characterized and established the subintestinal vein (SIV) plexus as a new model for vascular pruning.

The SIV is a blood vessel network that delivers blood to the digestive tract of the zebrafish larva. The SIV grows over the yolk sack, ventral to the posterior cardinal vein (PCV) and posterior to the common cardinal vein (CCV) [15]. Here, we apply high-resolution time-lapse microscopy techniques and several transgenic reporter lines to analyze the formation and the remodeling of the SIV plexus in developing zebrafish embryos. We show that different cellular processes such as single cell sprouting, vessel fusion, and vessel pruning are involved and timely coordinated to give the plexus its final shape. We describe in detail the cellular activities underlying different modes of vessel pruning, which are achieved through a variety of cell rearrangements and cell shape changes. The choreography of these processes during pruning resembles vessel fusion “in reverse” and can follow two different pathways determined by the flow environment in the pruning branch.

## Results

### SPIM Imaging Reveals Different Angiogenic Processes Involved in SIV Development

The plexus of the SIV emerges from the PCV ~2 days post-fertilization (dpf) as a reticular structure, which spreads bilaterally over the surface of the yolk [15]. By ~5 dpf, it has formed a set of parallel vessels situated along the dorsoventral axis and connected on the dorsal side to the subintestinal artery (SIA); a large vessel on the ventral side of the SIV plexus collects blood from all the parallel SIV branches and brings it back towards the heart.

**Fig 1:**
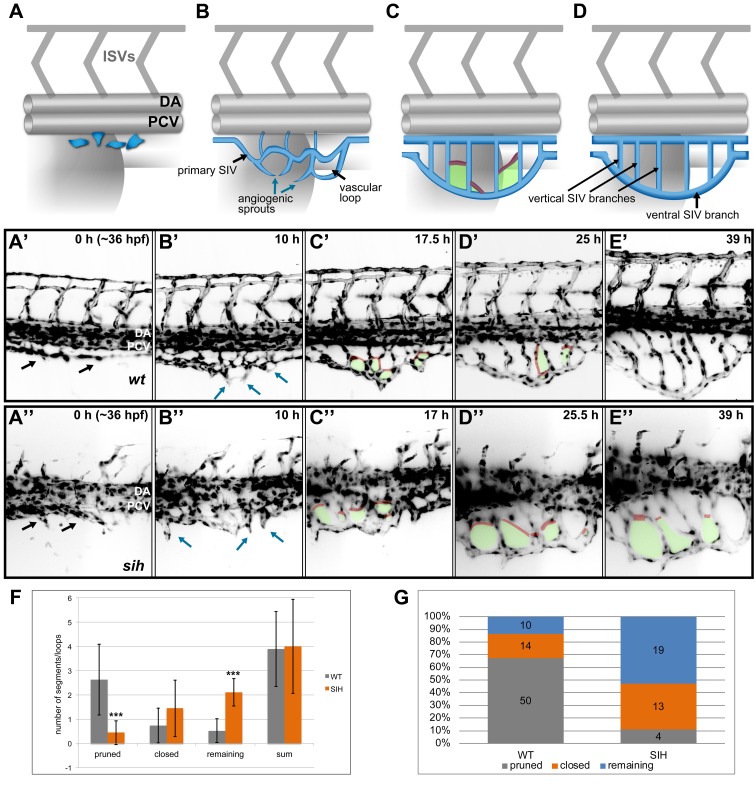
SIV development and maturation. **(**A–D) A model representing four phases of SIV plexus development in the zebrafish embryo between ~36 and 84 hpf. The SIV plexus is blue, and the dorsal aorta (DA), posterior cardinal vein (PCV), and intersegmental vessels are marked in grey. Single endothelial cells sprout ventrally and separate from the PCV (A) to form a primary SIV branch (B). Angiogenic sprouts grow out of the primary SIV and fuse to each other, forming a reticular plexus with multiple cross branches (C, red) and vascular loops (C, green); simultaneously, the plexus grows and moves ventrally. Eventually, the cross branches (and hence the loops) are removed, and the plexus simplifies, forming a set of parallel vertical branches draining into a large ventral SIV branch (D). (A’–E’) Stills from a SPIM time-lapse movie representing five phases of SIV development corresponding to models in A. (A”–E”) Stills from a SPIM time-lapse movie representing SIV development in a *silent heart* morphant embryo corresponding to models in A. In this case, the SIV keeps its reticular structure because of impaired pruning. (F) A graph comparing the SIV vascular loop formation and remodeling in a wild-type (grey) and silent heart embryo (orange), based on SPIM time-lapse experiments between 36 and 84 hpf. From the left, showing the number of cross branches pruned during remodeling phase, the number of cross branches/loops closed via collateral fusion, the number of cross branches/loops remaining until the end of the movie, and the sum of all loops observed throughout the movie. The values are average numbers per SIV plexus with standard deviation (*n* = 19 for wild type [WT] and *n* = 9 for *silent heart* [SIH]). *** *p* < 0.001. (G) A graph showing the percentage contribution of pruned (grey), closed by collateral fusion (orange), and remaining (blue) vascular loops to all events observed in WT versus *silent heart* embryos. See also S1 and S2 Figs, S1–S4 Movies, S1 Table and S1 Data.

Because a detailed description of SIV formation is missing, we followed the development of the entire plexus over several days by using Selective Plane Illumination Microscopy (SPIM), a novel technique for fast, long-term imaging of large fields of view [16]. In SPIM, images from different angles are acquired by rotating the sample, which is particularly beneficial for the large and curved structure of the SIV. Furthermore, the noninvasive embryo mounting [17] combined with low phototoxicity minimize interference of the imaging with the development of the vasculature [18]. We imaged Tg(*fli1a*:EGFP)^y1^ transgenic zebrafish embryos over 48 h (from ~36 to ~84 hours post-fertilization [hpf]) (S1 Movie and S2 Movie and Fig 1A–1E’). Based on these observations, we can divide the development of the SIV into four phases: (I) formation of the primary SIV tube through ventral sprouting of endothelial cells from the PCV followed by cell coalescence (Fig 1A–1A’, see S1A Fig and S3 Movie for details); (II) vascular loop formation through fusion of angiogenic sprouts originating from the primary SIV (Fig 1B–1B’, see S1B Fig and S4 Movie for details); (III) formation of a reticular structure with multiple vascular loops (Fig 1C–1C’); and (IV) remodeling into the final structure with parallel, vertical branches that drain into the large ventral SIV (Fig 1D–1E’).

The transition from phase III to IV involves extensive remodeling of the reticular structure, leading to a reduction of the number of loops and a redirection of the flow to the major vertical branches. Elimination of vascular loops occurs through regression of supernumerary cross branches or by collateral fusion of a cross branch to a neighboring major branch (S2 Fig and S5 Movie). Because of variability in the sprouting phase, the number of loops formed and the number of pruning events vary from embryo to embryo. To estimate the average number of loops, we analyzed 19 SPIM movies (each 40–50-h long) and quantified the number of pruned, closed by collateral fusion, and remaining loops as well as the overall loop number in each SIV plexus (Fig 1F and S1 and S2 Movie). We observed a total of 74 loops, with an average of ~4 ±1.5 loops per plexus. Fifty (~67%) of the loops were eventually removed by regression of the cross branch, 14 (~20%) were closed by collateral fusion of the cross branch to a neighboring major branch, and 10 (~13%) remained until the end of the monitoring time. From these results, we conclude that blood vessel regression is the preferred pruning mechanism during plexus remodeling in the SIV.

To determine whether blood flow is important for remodeling of the SIV plexus, we analyzed embryos injected at the single cell stage with the *tnnt2*/*silent heart* (*sih*) morpholino [19], which leads to a lack of heart beat and blood flow during later development (Fig 1A”–1E”). Compared to control embryos, *sih* morphants do not differ significantly in the number of loops formed (36 loops in nine movies, with an average of 4 ±2 per SIV plexus), indicating that the outgrowth and the sprouting phases do not require blood flow. Nevertheless, the remodeling of the plexus was strongly affected by the lack of flow and only 4 (11%) of cross branches regressed, whereas 13 (36%) were removed by vessel collateral fusion and 19 (53%) remained until the end of the observed period (compared to 13% in wild-type embryos) (Fig 1F and Fig 1G and S1 Table).

**Fig 2:**
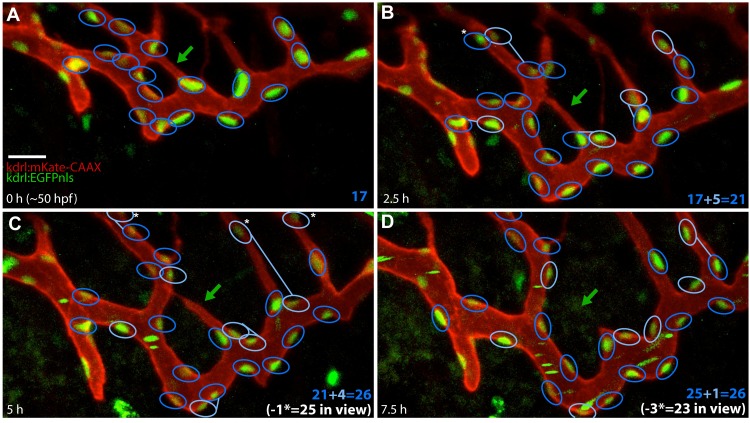
Analyses of endothelial cell nuclei during pruning. Analyses of the nuclei number and behavior in double transgenic embryos Tg(BAC:*kdrl*:mKate2-CAAX)^UBS16^; Tg(*kdrl*:EGFPnls) ^UBS1^. Nuclei were marked and quantified within a region of interest around the pruning segments (nuclei are encircled in dark blue, and green arrows point to a regressing branch). New nuclei derived from cell divisions happening between the time points are marked with light-blue circles and connected to the mother nuclei with a blue line (B–D). Nuclei that leave the field of view in the next time point are marked with asterisks (B–D). The nuclei changed positions in a way corresponding to cell rearrangements. The number of nuclei increased over time because of cell divisions. Nuclei numbers are indicated in the bottom right corner: in dark blue is the number of counted nuclei, in light blue the number of new nuclei (B–D), and in white the nuclei in view. No apoptotic nuclei were observed. See also S6 Movie and S2 Table. Scale bar: 20 μm.

Previous reports suggest that in certain cases pruning is accompanied by endothelial cell apoptosis [12,20]. We used a transgenic line labeling cell nuclei to verify whether apoptotic cells are present during the SIV plexus remodeling. In our time-lapse movies (S6 Movie, *n* = 10), we did not observe dying cells near the pruning vessels, and we noted an increase in nuclei number in analyzed pruning regions—a result of observed cell divisions (Fig 2 and S2 Table). The nuclei movements suggested that cell rearrangements, rather than apoptosis, account for vessel regression. The nuclei moved away from the pruning segments and incorporated into the remaining major SIV branches (Fig 2 and S6 Movie).

### Anastomosis in SIV Development Is Similar to Anastomosis in Other Vascular Structures

We have previously described vessel sprouts anastomosis in different vascular beds and have defined a highly stereotypic multistep process, which can follow one of two modes depending on either the presence or the absence of blood flow in the fusing sprouts [13,14].

To investigate whether these two modes of vessel anastomosis also occur during the formation of the SIV, we performed live imaging of early SIV development in a transgenic line, (Tg(*fliep*:GFF)^ubs3^,(UAS:mRFP),(*5xUAS*:*cdh5-EGFP*)^ubs12^), which labels cell–cell junctions by expressing green fluorescent protein (GFP) fused to a “tailless” version of vascular endothelial (VE)-cadherin. We observed that SIV sprouts follow the fusion steps as previously described. The early sprouts were not lumenized and formed anastomoses in a nonperfused environment. After the flow entered the developing SIV (~40 hpf), sprouts became lumenized and followed the steps of anastomosis previously observed in a perfused environment, including the formation of seamless tubes and the subsequent transformation into a multicellular tube (S1B Fig and S1C Fig and S4 Movie).

### Pruning Involves Cellular Rearrangements Similar to Anastomosis but in “Reverse” Order

Maturation of the SIV requires remodeling of the initial complex network in order to reach its mature topology, consisting of vertical, parallel branches connecting dorsal and ventral longitudinal vessels. In our long-term SPIM-imaging analyses, we found that remodeling is mostly achieved through regression of supernumerary branches formed during the SIV sprouting phase. The significant number of pruning events in each SIV plexus (2.5 ± 1.5, *n* = 19) and the convenient positioning of the SIV on top of the yolk allowed us to perform single cell analyses in great detail, using transgenic lines labeling cell–cell junctions (S7 Movie).

We found that, similarly to vessel fusion, pruning occurs in two variations characterized by the absence or presence of flow in the pruning branches during cell rearrangements, which we named pruning type I and II, respectively.

### Type I Pruning: Lumen Collapse before Cell Rearrangements

**Fig 3:**
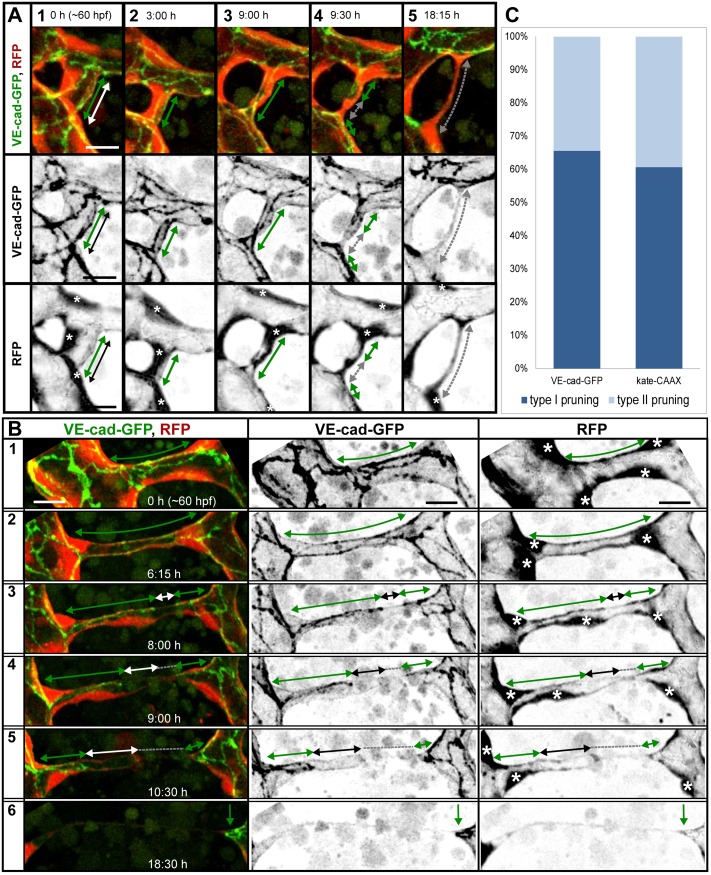
Cell rearrangements during pruning of type I and II. Stills from time-lapse movies illustrating cell rearrangements in type I pruning with lumen collapse before cell rearrangements (A) and type II pruning with cell rearrangements before lumen collapse (B) in transgenic embryos Tg(*fliep*:GFF)^ubs3^,(UAS:mRFP),(*5xUAS*:*cdh5-EGFP*)^ubs12^. Cell–cell junctions are green (VE-cad-EGFP), and cell cytoplasm is red. Black-and-white pictures show respective channels alone. Key steps of pruning are shown. Green arrows mark multicellular contacts (cell–cell junction length), white arrows mark transcellular lumen, and grey dotted lines mark unicellular fragments without lumen. Asterisks mark nuclei of cells contributing to the branch. (A) Pruning type I. A small, lumenized branch is made of two cells connected by two parallel lines of junctions (1). Lumen collapses when the branch is still multicellular (2–3); after lumen collapse, cells move away from each other, and cell–cell contact surface shrinks, generating a nonlumenized, ajunctional segment (4). Eventually, only the last bridging cell remains (5, grey arrow) prior to final detachment (not shown). See also S8 and S9 Movies and S3 Fig. (B) Pruning type II. Cellular architecture of a multicellular branch (1) is simplified to a branch made mainly by two cells connected by parallel lines of junctions (2). Further cell rearrangements lead to the formation of a partially unicellular tube (3). The lumen eventually collapses (4), and the cell body migrates towards the left-side major branch (5) until only a last, narrow cell extension connects two major branches (6). See also S10 Movie. (C) A graph representing percentage of pruning type I (dark blue) and II (light blue) in all pruning events analyzed in transgenic embryos of Tg(*fliep*:GFF)^ubs3^,(UAS:mRFP),(*5xUAS*:*cdh5-EGFP*)^ubs12^ and TgBAC(*kdrl*:mKate-CAAX)^ubs16^, respectively. Scale bar: 10 μm.

In pruning type I, the first recognizable step was the collapse of lumen in the vessel branch, which was transformed this way into a nonlumenized, multicellular cord with continuous junctional connections, often visible as two parallel lines (Fig 3A1–3A3 and S8 Movie). Subsequently, cells moved away from the pruning branch and incorporated into the neighboring major branches, eventually leaving a single bridging cell in between the latter (Fig 3A3 and Fig 3A4). This way, the vessel architecture changed from multicellular to unicellular, as visualized by changes in the junctional pattern. Initially, the junctions were continuous (Fig 3A1–3A3, green arrow), and after cell rearrangements, we observed ajunctional areas indicating unicellular vessel segments (Fig 3A4, grey arrow). During this transformation, the junctions of the last bridging cell changed from continuous along the cell body into two separate junctional contacts on both poles of the cell. With those junctions, the bridging cell was connected to the opposing major branches (Fig 3A4). Eventually, one of these connections shrank to a single cytoplasmic extension when the cell body migrated and incorporated into the opposing major branch (Fig 3A5 and S8 Movie). Finally, this last contact was resolved and regression completed (S3 Fig and S9 Movie).

### Type II Pruning: Cell Rearrangements before Lumen Collapse

In pruning type II, similar cellular rearrangements were observed, but the lumen was maintained in the process almost until the end. Initially, the junctions were continuous in the lumenized tube (Fig 3B1 and Fig 3B2, green arrow). Cell rearrangements were reflected in the change of the junctional pattern, and ajunctional areas appeared when only a single, bridging cell remained in the regressing branch (Fig 3B3 and Fig 3B4, white arrow). In this case, since the lumen was maintained, the last bridging endothelial cell transformed its architecture into a unicellular tube (Fig 3B3). The transcellular lumen of the unicellular tube collapsed, and the continuous apical surface within the cell was separated into two compartments (Fig 3B4). After the lumen collapsed completely, the cell body moved towards one of the major branches, shrinking its contact surface with the opposite major branch to a single spot (Fig 3B5 and 3B6). This last contact was eventually resolved as the cell completely incorporated into the opposite major branch (Fig 3B6 and S10 Movie).

We analyzed a total of 57 pruning events in transgenic lines marking endothelial cell–cell junctions and cell cytoplasm (Tg(*fliep*:GFF)^ubs3^,(UAS:mRFP),(*5xUAS*:*cdh5-EGFP*)^ubs12^, *n* = 29, S7 Movie) or cell membrane (TgBAC(*kdrl*:mKate-CAAX)^ubs16^, *n* = 28, S11 Movie). In the first case, we were able to assess both the cell arrangements and the presence of lumen during blood vessel regression. By labeling endothelial cell membranes with mKate2-CAAX, we were able to follow the dynamics of apical membrane, which is labeled more strongly than basal membrane and highlights inflated luminal compartments in unicellular blood vessels and cell–cell contact surface of multicellular vessels (S4 Fig and S11 Movie) [14]. From these experiments, we conclude that at least 30% of the pruning events involved formation of transient unicellular, lumenized tubes (Fig 3C).

### Multicellular to Unicellular Tube Transformation Is Mediated by Cell Self-Fusion

**Fig 4:**
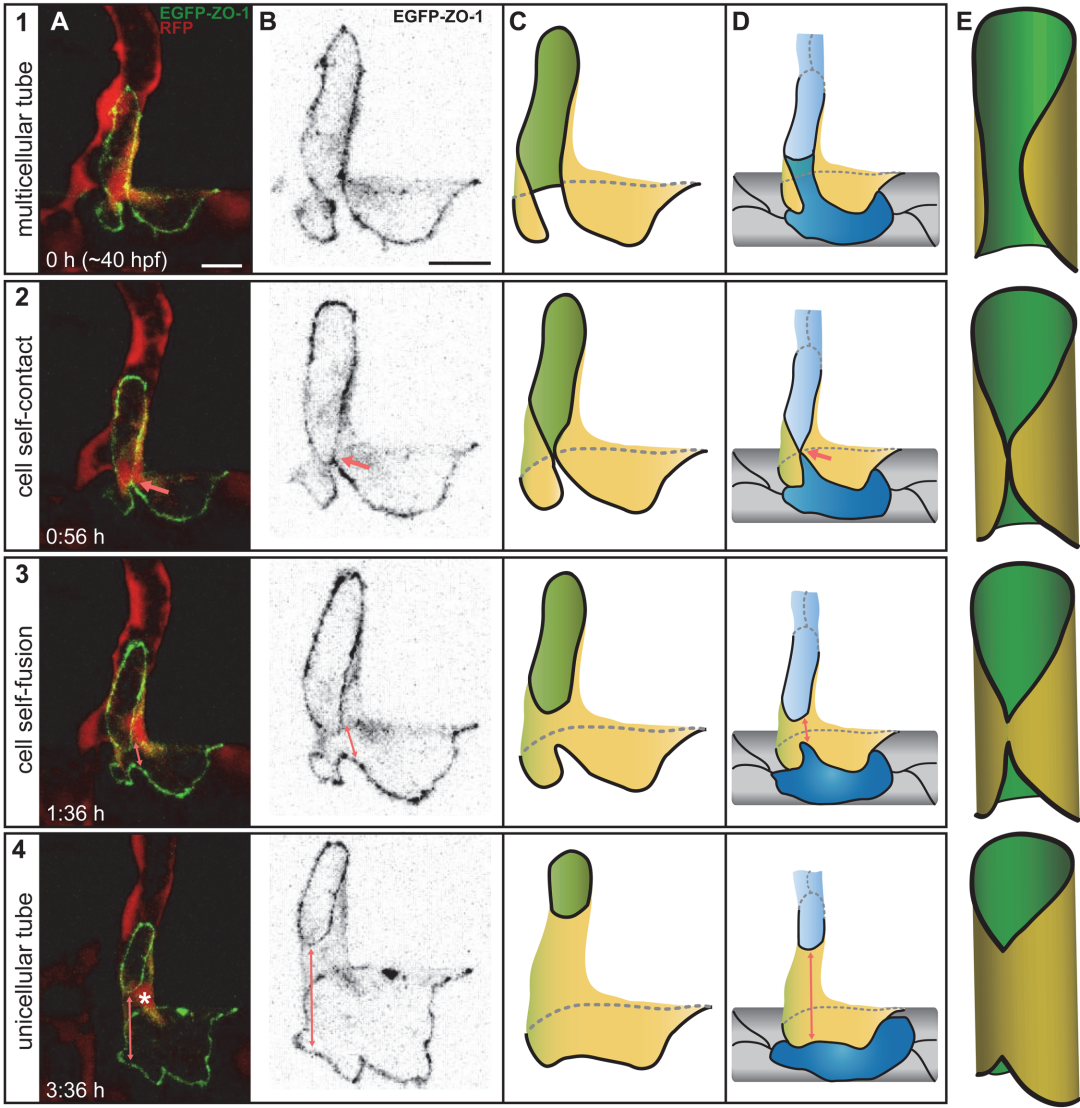
The mechanism of cell self-fusion. Stills from a time-lapse movie illustrating cell self-fusion during pruning of an arterial connection in the formation of a segmental vein in the fish trunk at ~40 hpf in a transgenic embryo Tg(*fliep*:GFF)^ubs3^,(UAS:mRFP),(UAS:EGFP-ZO-1)^ubs5^. Cell–cell junctions are green, and cell cytoplasm is red (A). Only the self-fusing cell is labeled with EGFP-ZO-1; the green channel alone is shown in B and modeled in C. The model in D shows all cells in the branch. E shows a simplified 3-D model of self-fusion: the junctions are black, the apical/luminal membrane is green, and the outer/basal membrane is yellow. The labeled cell is part of a multicellular tube (1). The cell reaches around the lumen and establishes a self-contact (2, arrow) when the neighboring cells move away from each other (blue cells in D). The cell expands the self-contact (3, arrow), and its membranes fuse, as no junctional connection is visible along the tube. Two junctional rings connect the cell to its neighbors: to the intersegmental vessel (ISV) on top and to the dorsal aorta at the bottom. The cell forms a funnel-shaped unicellular tube (4). The arrow shows the tube length, and the asterisk marks the nucleus. Scale bars: 10 μm. See also S12 and S13 Movie.

Our time-lapse analyses revealed that type II pruning involves the formation of transient unicellular tubes formed by a single endothelial cell, which wraps itself around the lumen. Surprisingly, upon contact with its contralateral side the cell starts to self-fuse its cell membrane in a zipper-like fashion, thereby transforming the cell into a seamless, unicellular tube with a transcellular lumen (Fig 4). To demonstrate endothelial cell self-fusion more directly, we performed single cell labeling experiments with the junctional marker EGFP-ZO-1 (Tg(*fliep*:GFF)^ubs3^,(UAS:mRFP),(UAS:EGFP-ZO-1)^ubs5^), which tends to be expressed in a mosaic fashion [13]. Since this transgene is hardly active in venous vessels, we examined whether endothelial cell self-fusion occurs during regression of segmental arteries (SeAs), which takes place during segmental vein formation (SeV) in the trunk. SeVs sprout from the posterior cardinal vein and form by anastomosis with SeA, thereby transforming the latter into SeVs [21]. This fusion event is accompanied with the regression of the proximal segment of the SeA (S5 Fig and S12 Movie). Using the UAS:EGFP-ZO1 marker, we were able to follow a single endothelial cell as it formed a unicellular tube (Fig 4 and S13 Movie). The junctional transformation of this cell evidenced by ZO-1 expression was entirely consistent with endothelial cell self-fusion. Initially, the SeA was connected to the dorsal aorta with at least two cells, one of them labeled with EGFP-ZO-1 (Fig 4A, green cell). The arterial segment, initially multicellular, undergoes rearrangements similar to the ones described for the SIVs, also in two possible variations (S5 Fig). Fig 4 shows pruning type II, in which lumen was maintained during cell rearrangements and the remaining “last bridging” cell wrapped around the lumen and self-fused to form a unicellular tube. The cell had a funnel-like shape and connected the aorta (bottom, large junctional ring) to the SeA (top, small junctional ring). Eventually, the SeA connection to the aorta was resolved, and the cell incorporated completely into the aorta (S13 Movie).

**Fig 5:**
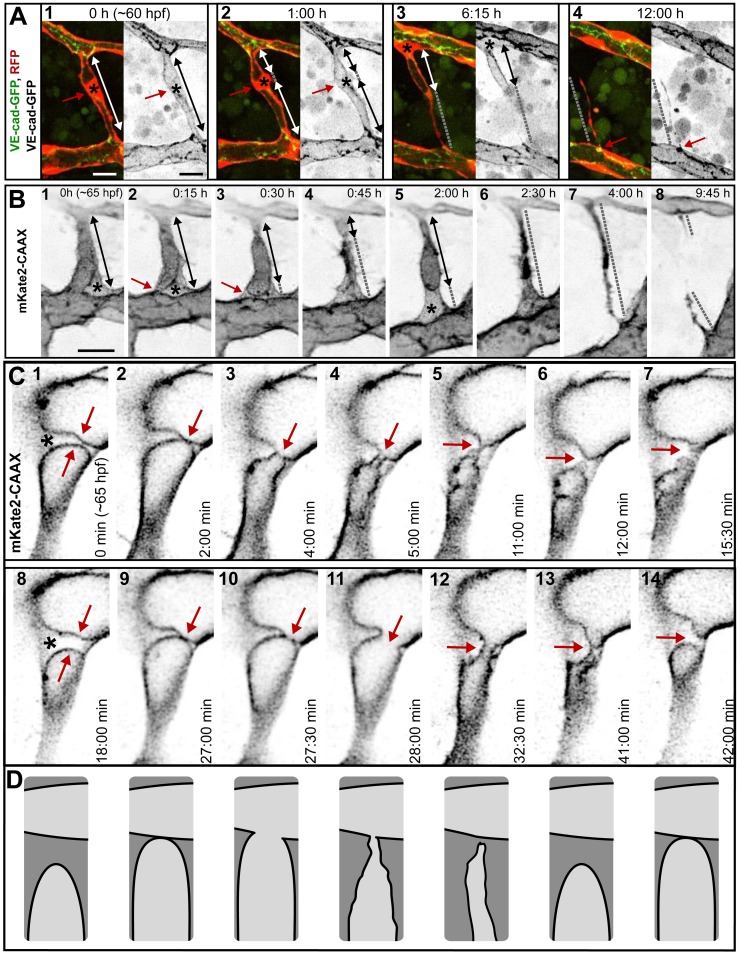
Dynamic of lumen collapse in a unicellular tube. (A) Stills from a time-lapse movie illustrating lumen collapse in a unicellular tube in a transgenic embryo Tg(*fliep*:GFF)^ubs3^,(UAS:mRFP), (*5xUAS*:*cdh5-EGFP*)^ubs12^. A single lumenized “last link” cell connects two major branches (1). The white/black arrow marks lumen length. The asterisk marks the nucleus; the red arrow points to the narrowest lumen part (next to the nucleus). The lumen splits first next to the nucleus (2, red arrow), forming two distinct luminal compartments within the cell (2, white arrows) that are separated by a nonlumenized part (grey dotted line). The nonlumenized part increases in length as the lower luminal compartment collapses (3). The cell body (nucleus, asterisk) moves towards the upper major branch. The last cell extension (gray line) contacts the lower major branch with a spot-like junction (4, arrow). (B) Stills from a time-lapse movie showing lumen collapse in a unicellular tube in a transgenic embryo TgBAC(*kdrl*:mKate-CAAX)^ubs16^. Black arrows show continuous lumen, gray dotted lines show nonlumenized unicellular regions, the red arrow shows the point of lumen breakage, and asterisks mark the nucleus, where clearly distinguishable. Lumen breaks at the contact site to the lower major branch, next to the nucleus (1–3). The luminal compartment deflates and inflates again (4–5, arrows). After complete lumen collapse, the last connection is resolved (6–8). See also S14 Movie. (C) Stills from a time-lapse movie showing lumen collapse in higher time resolution. Inflated luminal compartments (1, arrows) are framed by apical membrane (marked by mKate2-CAAX) and separated by a thin bridge of cell body, most likely the nucleus (1, asterisk). The lumen expands and two apical membranes touch (2, arrow) and fuse (3), but the lumen does not completely inflate (4). The lumen breaks again (5–7) and reconnects in a similar fashion (8–11) within a short time. See also S15 Movie. (D) A schematic representing luminal instability, based on still pictures in C. The apical membrane is black, the cell body is dark gray, and the lumen is light gray.

In type II regression, the vascular lumen collapses in a unicellular context. Here, the continuous apical compartment lining the luminal site of the cell was separated into two compartments when the opposing apical membranes collapsed on each other because of lumen deflation (Fig 5). Interestingly, in many cases the lumen split right next to the nucleus, where the cell body takes up the most space (Fig 5A and Fig 5C1, asterisks), thereby facilitating the separation of luminal compartments (Fig 5B and S14 Movie). Even though in several cases it took up to 12 hours to complete this step of the pruning process, the lumen collapse itself was very fast. When we analyzed luminal membrane using high-resolution time-lapse imaging, we found that the lumen broke and reconnected multiple times before completely separating the two remaining luminal compartments (Fig 5C and Fig 5D and S15 Movie).

## Discussion

### SIVs as a Novel Pruning Model in Zebrafish

Developmental pruning of vascular networks has been described in various vertebrate organs, most notably in the extraembryonic tissues of the avian embryo and the postnatal mouse [22]. Here, we describe the SIV plexus of the zebrafish embryo as a novel in vivo model to study angiogenesis and vascular remodeling. Using various transgenic reporters and cutting-edge microscopy techniques, we first describe vascular remodeling on a plexus-wide scale and then turn to the analysis of single cell behaviors to uncover the cellular mechanisms of blood vessel regression.

Vascular remodeling is an extremely dynamic process that is regulated by molecular as well as physical cues [6,7,23,24]. While the molecular regulation has been studied in mouse retina [6,7,24], avian chorion-allantoic blood vessels have been used to study the dynamics of vascular remodeling [9]. In zebrafish, the blood vessel regression has previously been studied in the mid-brain [11], the eye [12], and during intersegmental vessel formation in the trunk of the embryo [21]. In the latter two contexts, pruning appears to be triggered by a fusion event between an angiogenic sprout and an existing vessel. This fusion event generates a “T-junction,” which subdivides the existing vessel into two segments of different flow patterns, which ultimately lead to the regression of one of the segments. This is in contrast to the pruning patterns we observe in the SIV. Here, the blood vessels first form a reticular network and then remodel through pruning of supernumerary segments. This temporal separation of angiogenesis and pruning resembles formation of the murine retinal vasculature. In the retina, sprouting and fusion of vessels takes place at the angiogenic front, whereas pruning is observed in proximal regions [7,25]. Therefore, the SIV plexus of the zebrafish provides an in vivo model well suited to complement studies of vascular remodeling in the mouse retina, with the added benefit of accessibility for live imaging.

### Variation in the Timing of Lumen Collapse Generates Two Cellular Modes of Blood Vessel Pruning

In all instances that we observed, pruning involved extensive rearrangements of endothelial cells, which moved out of the branch and incorporated into the neighboring vessels. Furthermore, we did not observe cell death or hemorrhages during this process. In agreement with previous reports [11,12], we observed a reduction and eventually the halt of blood flow in pruning branches, prior to their disassembly. The lumen, however, was often maintained and persisted in endothelial remodeling up to the point when only a single cell connection was remaining. In fact, the choice between the two morphogenetic pruning pathways (multicellular versus unicellular) correlated with the timing of lumen collapse relative to the initiation of the cell rearrangements. This suggests that endothelial cells adapt their morphogenetic behavior to the constraints imposed by the state of the vascular lumen at the onset of blood vessel pruning.

### Physical and Molecular Regulation of Blood Vessel Fusion

Based on our observations, the selection of the pruning mode I or II is a consequence of the cellular topology and flow environment of the pruning segment. The primary question is how the segment to be pruned is selected in the first place. Genetic factors such as Notch/Dll4 [6] and Wnt [26] have been shown to influence vessel remodeling in the developing mouse retina. Importantly, reduction in Notch/Dll4 signaling led to an up-regulation of vasodilators such as adrenomedullin and prevented retinal capillary regression [6], thus suggesting that Notch/Delta signaling may provide the switch for the selection of pruning blood vessels by locally modulating blood flow patterns. The role of Wnt signaling in blood vessel pruning is less clear. While several Wnt ligands and receptors have been implicated in blood vessel regression [26], a recent study by Korn et al. [24] has investigated the role of noncanonical Wnt signaling by endothelial specific knock-out of Evi, which is required for the secretion of Wnt ligands. Such Evi knock-out retinas not only display strongly enhanced blood vessel regression in the proximal (postangiogenic) region of the retina but also affect blood vessel density at the angiogenic front. Furthermore, and in contrast to our observations, these defects could be attributed to a decrease in endothelial cell proliferation and an increase in apoptosis. However, recent studies on blood vessel regression in the retina (see accompanying paper by Franco et al. [27]) as well as our observations do not support apoptosis as a key regulator of blood vessel regression. These discrepancies suggest that noncanonical Wnt signaling is not directly involved in blood vessel selection for pruning.

The observation that changes in blood flow patterns precede blood vessel regression suggests that mechanical forces are essential regulators of pruning. In agreement with this notion, blood pressure, shear stress, and flow type were shown to affect vessel remodeling [28,29]. When the flow was disrupted, vessels did not remodel properly. Interestingly, the remodeling involved detachment of small branches defined by differences in flow and the angle of branch bifurcation [30]. Also in the zebrafish brain vessels, pruning patterns could be altered by changes in blood pressure. Pruning was induced in branches with artificially halted flow and inhibited when blood pressure was chemically raised [11]. In the mouse yolk sac vasculature, erythroblast circulation was required for remodeling and for the expression of force-regulated factors, pointing at the role of blood viscosity [10]. Our results show that in the SIV mostly the small, bifurcated branches are removed and that instable blood flow and lumen are the first signs of pruning, suggesting that a similar flow-dependent selection mechanism could be involved. This view is supported by our observation that pruning in the SIV is impaired in the absence of blood flow, suggesting that certain flow-dependent factors, likely the differences in pressure between branches, trigger pruning.

**Fig 6:**
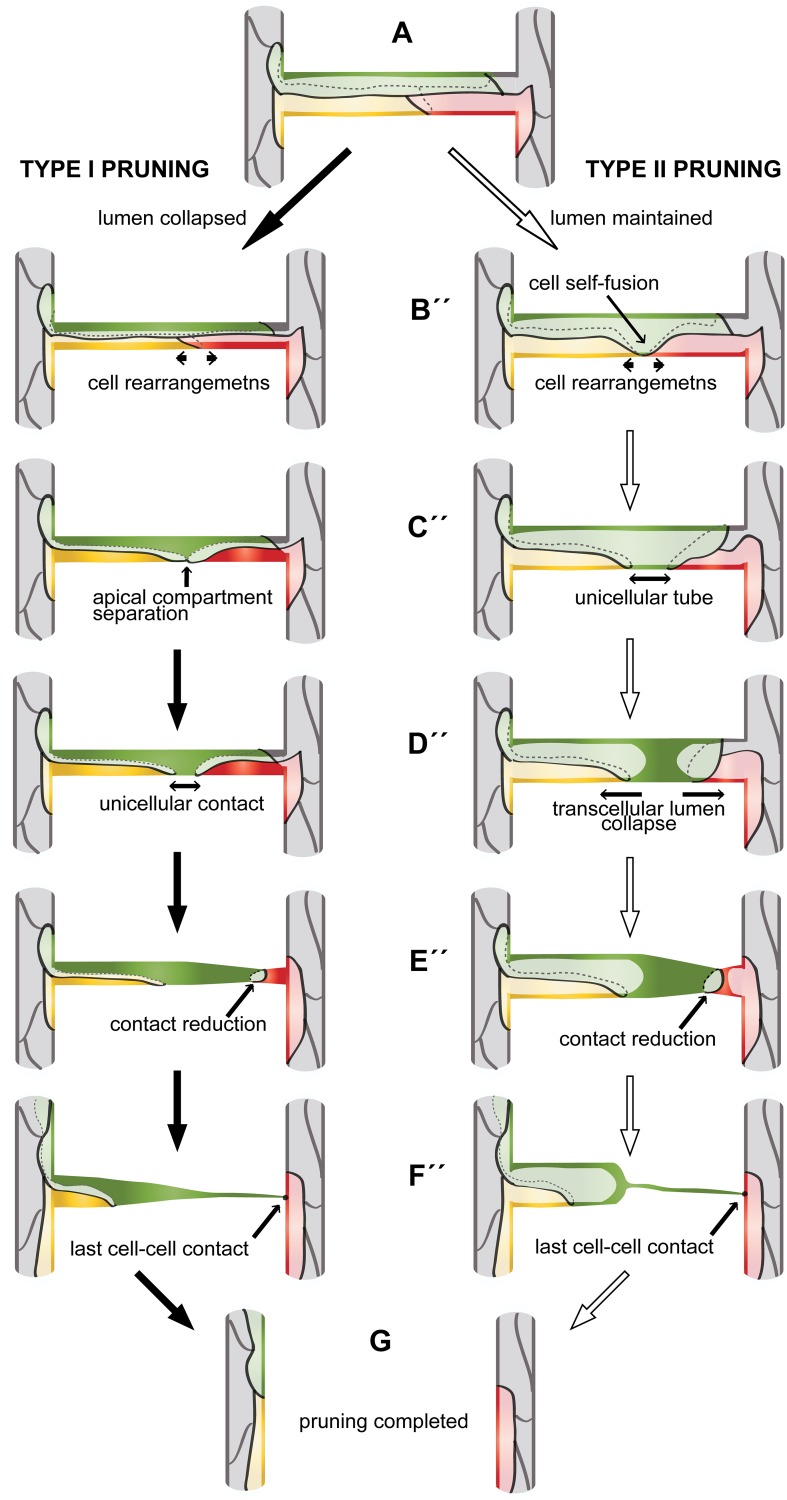
Two cellular models of vascular pruning. A pruning branch is initially a multicellular tube (A). The cellular rearrangements to follow depend on collapse or maintenance of lumen at this stage (pruning type I or II, respectively). If the lumen collapses before cell rearrangements (type I pruning, B’), cell rearrangements lead to formation of a unicellular connection (C’–D’). The last linking cell regresses (E’) and completely resolves the last connection (F’) to complete the pruning process (G). If the lumen is maintained, cell rearrangements lead most cells out of the branch (B‘‘, arrows) and force the remaining cell to undergo self-fusion and form a unicellular tube (C”, arrow). Transcellular lumen collapses in the unicellular tube, forming two separate luminal compartments (D”, arrows). The last cell reduces its contact to one of the major branches (E”) and eventually the last contact (F”) is resolved and pruning is complete (G). See also S6 Fig.

Upon initiation of blood vessel regression, endothelial cells migrate out of the regressing branch. This behavior is regulated by a change in cell polarity, which itself appears to be controlled by hemodynamic forces (see accompanying article [27]). Our analyses are in agreement with this view and show that these cell rearrangements and especially the lumen collapse are highly dynamic processes that are strongly influenced by the presence of blood pressure in the developing vessels. During SIV pruning, endothelial cells can embark on two different morphogenetic pathways, of which only type II pruning involves cell wrapping and cell self-fusion (Fig 6). At this point, we do not have evidence whether a particular signaling pathway regulates the choice of the morphogenetic pathway. Type I and type II pruning mechanisms split up only after endothelial cells have initiated cell rearrangements, suggesting that neither Notch/Dll nor noncanonical Wnt signaling is directly involved in the determination of type I versus type II specification. In contrast, type I/II pruning strongly correlates with the presence (type II) or absence (type I) of lumen during this process. This correlation suggests that pruning type selection is rather controlled by mechanical cues and that endothelial cells adapt their behavior to different luminal topologies during cell rearrangement.

### Vessel Remodeling through Collateral Fusion Resembles Reversed Intussusception

Apart from blood vessel regression through pruning, we observed an alternative mechanism for reducing the number of vascular loops, namely collateral fusion. Morphologically, collateral fusion resembles reversed blood vessel intussusception, which is an angiogenic process in which vessel plexus expands by splitting existing branches along the longitudinal axis. Intussusception is induced in response to high flow and therewith-increased wall shear stress [28,31]. Interestingly, in the SIVs we observed an increase of collateral fusion events in the embryos lacking blood flow and hence pruning. Further studies will show whether these processes are regulated and coordinated by the blood-flow-related factors.

### Pruning as Fusion in Reverse

In this study, we demonstrate that blood vessel pruning is a stereotyped, stepwise process. On a cellular level, vessel pruning resembles vessel fusion, but many of the cell activities happen in a reverse order. However, the molecular mechanisms employed for certain cellular activities in a reverse order should be quite different. In both fusion and pruning, we observed changes in cell polarity, dynamic formation and resorption of apical/luminal membrane, and dynamic cell rearrangements that impose dramatic shape changes on endothelial cell architecture. Interestingly, the presence or absence of luminal pressure defines the mode of blood vessel fusion as well as pruning.

A critical step during blood vessel fusion is the formation of a continuous lumen, which can happen either through cell rearrangements and formation of a multicellular cord (in a nonperfused environment) or through pressure-dependent apical membrane invagination and formation of a seamless unicellular tube (in a perfused environment) [13,14]. Analogously, during vessel pruning, we observe a reversed process in which a continuous luminal compartment within the “last bridging” cell is partitioned into two separate ones. Also in this case, we observed two different scenarios. In type I pruning, analogous to fusion in a nonperfused environment, cell rearrangements following lumen deflation lead to the subdivision of the continuous apical compartment of the last linking cell into two individual compartments. This becomes apparent when continuous junctions are separated into two independent rings on the opposing ends of the cell (Fig 6B’–6D’). In type II pruning, analogous to fusion in a perfused environment, a seamless tube forms as a result of cell rearrangements; subsequently, the lumen collapses within this unicellular tube, leading to fission of apical membranes and generation of two separate luminal compartments (Fig 6C”–6 D” and S6 Fig).

During anastomosis as well as during regression, a blood vessel often goes through a transient unicellular state, which is marked by unsteady and intermittent blood flow. During this phase, the luminal/apical compartment collapses (forming transient vacuole-like structures) and reforms in rapid succession. On the one hand, this shows that continuous blood pressure is required to stabilize the luminal compartment within a unicellular tube. On the other hand, the reiterative splitting and rejoining of apical membrane compartments within endothelial cells illustrates an extensive capacity of apical membrane for budding and fusion—presumably mediated by factors implicated in vesicle trafficking and fusion [32].

### Cell Splitting and Cell Self-Fusion: Two Faces of the Same Coin?

During the pruning process, we find that the transformation of a multicellular tube into a unicellular tube results from one endothelial cell wrapping around the lumen and establishing a self-contact, which ultimately results in membrane fusion at the lateral side of the cell and formation of a seamless tube. This process of cell self-fusion resembles cell splitting in reversed order, a process we have previously described as occurring during blood vessel fusion, in which an initial unicellular tube transforms into a multicellular tube and the cell shape undergoes a reversed transformation, from a seamless tube cell into a flat cell [14]. Even though geometrically similar and reversed, these two processes must involve very different molecular mechanisms, since different membrane compartments interact with each other in each case (S6 Fig). During cell splitting, two inner leaflets of opposing lateral membranes approach each other and must be the first to fuse. This process could be mediated by intracellular components, for example, similar to those involved in vesicle fusion or those involved in cell abscission—the final step of cytokinesis [33]. In cell self-fusion, a cell wraps itself around a lumen until the two outer membrane leaflets are connecting. Such a cell behavior has been previously described in the tracheal system of Drosophila [34]. Here, cell wrapping results in a unicellular tube sealed by an autocellular adherens junction. During endothelial cell wrapping, an initial autocellular contact is made, but it does not result in an extensive autocellular junction. Instead, the cell–cell contact is followed by membrane fusion. It will be interesting to decipher the molecular mechanisms that allow or prevent cell self-fusion in endothelial and tracheal cells, respectively.

Cell fusion is a rather prominent occurrence during development—for instance, it occurs in the fusion of gametes, the generation of multinucleate muscles, and placenta formation [35]. Cell self-fusion is a much more rare phenomenon but has already been described in the formation of tubular structures in *Caenorhabditis elegans* [36,37]. During the formation of the digestive system, several cells autofuse to generate toroid structures, similar to what we observed during vascular pruning. In the case of the *C*. *elegans* intestinal tract, autofusion (self-fusion) is mediated by so-called fusogens (AFF-1 and EFF-1, respectively), which help to bring adjacent membranes in close proximity to initiate membrane fusion [36]. It remains to be investigated whether proteins with similar function exist in vertebrates.

Cell self-contact elimination (similar or identical to cell self-fusion) has recently been reported in epithelial Madin-Darby canine kidney (MDCK) cells plated on a micropillar array [38]. In this assay system, cells were forced to grow around the pillars to take up a torus shape, with the pillar in the middle, and to make contacts with themselves at the distal side of the pillar. Such cell self-contacts resulted in cell self-fusion, promoted by the presence of E-cadherin, which brings cell membranes into proximity and thus facilitates membrane fusion. Strikingly, cells lacking E-cadherins, such as fibroblasts, failed to self-fuse. This suggests that cell self-fusion is an inherent feature of epithelial cells that have to ensure continuity of the tubular organ lining.

To the best of our knowledge, our study is the first report demonstrating that cell self-fusion is an integral part of the complex morphogenetic process during vascular pruning in vertebrates.

## Materials and Methods

### Ethics Statement

The animal experiments were approved by the Kantonales Veterinaeramt Basel-Stadt (license number 1995/1996) and in accordance with EU directive 2011/63/EU as well as the German Animal Welfare Act.

### Fish Maintenance and Stocks

Zebrafish were maintained at standard conditions [39]. Embryos were staged by hpf at 28.5°C [40]. The following zebrafish lines were used in this study: Tg(*fli1a*:EGFP)^y1^ [41], Tg(*kdrl*:EGFP)^S843^ [42], Tg(*kdrl*:EGFPnls)^UBS1^ [43], Tg(UAS:EGFP-ZO1-cmlc:EGFP)^UBS5-7^ [13], Tg(UAS:RFP) and Tg(*fli1ep*:GAL4FF)^UBS2-4^ [44,45], Tg(*5xUAS*:*cdh5-EGFP*)^ubs12^ [14], Tg(BAC:*kdrl*:mKate2-CAAX)^UBS16^ [14], and Tg(UAS:Kaede)^rk7^ [46].

*Tnnt2/silent heart* (*sih*) Morpholino injection was performed as described before [19].

### In Vivo Time-Lapse Analysis Using Confocal Microscopy

Transgenic embryos selected for presence of fluorescence were anaesthetized in 1x tricaine (0.08%) and mounted in a 35-mm glass-bottom petri dish (0.17 mm, MatTek), using 0.7% low-melting agarose (Sigma) containing 0.08% tricaine and 0.003% PTU. A Leica TCS SP5 confocal microscope was used for time-lapse analyses. Images were taken using the following objectives: 20x air or 40x water immersion. High time-resolution analyses were performed using a Perkin Elmer Ultraview spinning disc microscope and a 63x water immersion objective, and images were deconvolved using Huygens Remote Manager software [47]. All images are maximum intensity *z-*projections. Photoconversion of the Kaede protein was performed as described before [13]. The posterior cardinal vein was selected as a region of interest and illuminated with a 405-nm solid-state laser for ~30–40 s to achieve different color labeling of the venous (red) and arterial (green) cells.

### In Vivo Time-Lapse Analysis Using SPIM and Image Processing

To avoid pigmentation, transgenic zebrafish embryos were kept in 0.2-mM N-Phenylthiourea (PTU) from 14 hpf until 35 hpf. At 35 hpf, transgenic zebrafish embryos were selected for the presence of fli1a:EGFP fluorescence and mounted in fluorinated propylene ethylene (FEP) tubes, which had been coated with 3% methyl cellulose and filled with 0.1% low-melting agarose [48]. To prevent movement of the fish, the chamber of the SPIM setup was filled with E3 fish medium containing 0.03% tricaine.

Long-term time-lapse imaging was performed on a home-built multidirectional SPIM (mSPIM) setup [49], equipped with a Leica HCX APO L 20x/0.50 W detection objective, a Coherent Sapphire 488-nm laser, and two Andor iXon 885. Both SIV plexuses of the embryo were imaged every 15 min for up to 60 h (S1 Table and S1 Data). The images were acquired from one angle for each SIV with an axial resolution (z-stack spacing) of 3 μm. To image the whole plexus length, several regions were acquired and stitched together using an adapted version of the stitching tool [50]. The acquired images were processed and background was subtracted using available and custom written Fiji plugins [51]. The side projections were generated using thresholding by Huang [52] and subsequent Chamfer Matching [53]. ISVs were used as a reference point to follow the embryo growth and adjust the side-profile absolute position over the time-lapse to achieve an optimal projection. To generate the 3-D rendering in S2 Movie, the 3-D project plugin (routine written by Michael Castle and Janice Keller of the University of Michigan Mental Health Research Institute [MHRI]) of ImageJ was applied using brightest point projection, interpolation, and angle increment of 10 degrees.

### Time-Lapse Data Quantification

The quantification of pruning events in long-term SPIM movies was performed on maximal projections. Vascular structures generated between one or two major branches and a small branch were counted as vascular loops. The number of pruned, closed by collateral fusion, and remaining loops was counted for each experiment throughout the time lapse (S1 Table). Average values were compared between wild type and *silent heart* experiments using unpaired, two-tailed Student’s *t* test (S1 Data).

The quantification of nuclei number in time-lapse movies was performed on maximal projections, within a region of interest defined around the pruning branches. Nuclei numbers were assessed every 10 time points before, during, and after pruning (S2 Table). Movies were analyzed qualitatively for the presence of apoptotic and dividing nuclei. Nuclei tracking in S6 Movie was performed using Imaris Spot Tracking tool (Bitplane).

## Supporting Information

S1 Data Quantification of pruning events in live-imaging experiments. The Excel file contains tables and quantifications that served to generate graphs in Fig 1F and Fig 1G (pruning events in the SIVs of wild-type and *silent heart* morphant embryos) and Fig 3C (pruning type I and type II). Each sheet contains the number of pruning events calculated for each imaging experiment, the statistical analyses, and the graphs generated from these results.
(XLSX)

S1 Fig SIV outgrowth involves single cell assembly and angiogenic sprout fusion. Stills of time-lapse movies showing outgrowth of the SIVs between ~36 and ~60 hpf in transgenic embryos: Tg(*fli1a*:EGFP) in A and Tg(*fliep*:GFF)^ubs3^,(UAS:mRFP), (*5xUAS*:*cdh5-EGFP*)^ubs12^ in B and C. (A) SIV emerges from the PCV at ~36 hpf (1–2). Single cells sprout ventrally from the PCV (2–3, arrows). The cells that contact each other (4, arrows) often lose contact with the PCV. A single, nonlumenized primary SIV forms and produces ventral angiogenic sprouts (5, arrows) and eventually lumenizes (6). PCV, posterior cardinal vein; DA, dorsal aorta; CCV, common cardinal vein; ISV, intersegmental vessel; DLAV, dorsal longitudinal anastomotic vessel. See also S3 Movie. (B) Multiple angiogenic sprouts originating from the primary SIV connect to each other (arrows) before (1–3) and after (4–6) lumen inflation, forming a net of vascular loops. (C) Close-up of SIV sprout fusion including the VE-cad-EGFP channel, showing new contact formation (2, arrow marks a spot of junctions), contact expansion (3, a ring of junctions), and transformation of the new branch into a multicellular tube (lines of junctions in 4). See also S4 Movie. Scale bars: 50 μm (A and B) and 10 μm (C).
(TIF)

S2 Fig Closing of a vascular loop by collateral vessel fusion. A small branch forms a vascular loop with the major branches (1, arrow). The opening of the loop shrinks as the small branch approaches the major branch sidewise (2–3, arrows). The last very small opening is closed as the side walls of two vessels touch (5) and eventually connect to form a single lumen (6). Fusing lumens of the branches are marked with blue arrows. Scale bars: 10 μm. See also S5 Movie.
(TIF)

S3 Fig Final steps of pruning type I. A small, lumenized branch is made of two cells (A). Lumen collapses when the branch is still multicellular (B); after lumen collapse, cells move away from each other, and the cell–cell contact surface shrinks (C). The last cell–cell contact (D, green arrow) is eventually resolved completely as the last cytoplasmic extention of the bridging cell detaches from the major branch (E). See also S9 Movie.
(TIF)

S4 Fig Analyses of pruning vessel with the cell membrane marker. The TgBAC(kdrl:mKate-CAAX) transgenic line allows visualization of the endothelial cell membranes. Transcellular lumen formation and collapse can be distinguished by the presence of inflated, round-ended, apical membrane compartments (black arrows). In certain cases, it is also possible to recognize multicellular vessel fragments by the increase in staining density at the cell–cell contact surfaces (green arrows). The nuclei are recognizable as round structures that are brighter because the two membranes (apical and basal) are well separated around the nucleus (red asterisks in the vessels of interest). These criteria were used for quantifications presented in Fig 3C. Three pruning vessel segments are followed in this figure. Segment 1: lumen in a unicellular vessel fragment (black arrow) is parted next to the nucleus (red asterisk). The lumen perfuses (B) and deflates again (C) to finally collapse completely (D) when the vessel detaches (E, dotted line). Segment 2: a multicellular vessel fragment with the cell–cell contacts visible as darker lines of the membrane staining (A–D, green arrows) narrows significantly when the lumen collapses (E–F). Cells leave the branch after the lumen collapse (D–F, asterisks mark the nuclei). Segment 3: a unicellular vessel fragment undergoes cell division generating two nuclei (C, asterisks), a new cell–cell contact surface (C, green arrow), and two luminal compartments (C, black arrows). The lumen is partially restored (D) but later collapses, leaving a small vacuole-like structure (E, black arrow). The last cell–cell contact surface is reduced as the pruning proceeds (E–F). See also S11 Movie.
(TIF)

S5 Fig Pruning in the ISVs. **(**A) Stills of a time-lapse movie showing segmental vein formation in a transgenic embryo Tg(fliep:GFF)^ubs4^; Tg(UAS:Kaede)^rk7^. Arterial cells are marked in red, venous cells are green (photoconverted Kaede, colors are inverted for better visualization). A new venous sprout (green arrow) grows out of the PCV towards a segmental vessel (red arrow). The venous sprout connects to the ISV. At the same time, the ISV segment connected to the aorta narrows and eventually detaches as the cells migrate up to contribute to the dorsal part of the ISV. See also S12 Movie. (B) Still images of the segmental vein formation in a transgenic embryo Tg(fliep:GFF)^ubs4^; Tg(UAS:mRFP);Tg(UAS:EGFP-ZO-1)^ubs5^. A single cell is expressing the EGFP-ZO-1 (green). The cell belongs to the ISV and is anchored in the DA (red arrow points to the ring-like junction). When the venous sprout connects (green arrow), the green cell moves up the ISV. The junctional ring gradually reduces in size to a spot, and the cell eventually detaches from the aorta and migrates up the ISV. See also S13 Movie. (C) A cellular model of pruning during the segmental vein formation. Arteries are grey, and venous cells are green. Red and blue cells are initially connected to the DA. As the venous sprout attaches to the ISV, the arterial cells detach from the DA and move up to contribute to the dorsal part of the ISV. (D) Stills from a time-lapse movie showing multicellular-to-unicellular tube transformation during pruning in an ISV in a transgenic embryo Tg(fliep:GFF)^ubs4^; Tg(UAS:mRFP);Tg(UAS:EGFP-ZO-1)^ubs5^. Key steps of pruning are shown, corresponding to the model in Fig 6. Green arrows mark multicellular contacts (cell–cell junction length), white arrows mark transcellular lumen, and grey dotted lines mark unicellular fragments without lumen.
(TIF)

S6 Fig Comparison of cell activities in vessel pruning and fusion. Cell rearrangements and shape and polarity changes happen in reversed order during vessel pruning (black writing), as compared to vessel fusion (red writing). (A–C) Formation of a new contact involves de novo apical polarization and expansion of a new junctional connection. A corresponding last pruning step involves shrinking and resolution of the junctional contact, which also implicates removal of the apical membrane compartment. (D) Expansion of apical membrane compartments leads to lumen coalescence and opening. The inversed process leads to lumen collapse and separation of a continuous apical compartment into two separate ones. (E) A unicellular tube is a transient structure that forms through invagination and fusion of the apical membrane (during vessel fusion) or through cell self-fusion (during pruning). (F–G) Cell self-fusion is accompanied by migration of neighboring cells away to opposite sides. Splitting of a unicellular tube, resembling a “reversed” self-fusion, is accompanied by neighboring cell migration towards the cell.
(TIF)

S1 Movie Development of the SIV plexus in wild-type and *silent heart* embryos. Related to Fig 1B and Fig 1C. Time-lapse movies showing the SIV plexus development in Tg(*fli1a*:EGFP)^y1^ embryos between ~36–94 hpf, in wild-type (up) and *silent heart* morpholino–injected embryo (down). The movies were acquired using a custom-built multidirectional SPIM (mSPIM). The side projections are shown in yellow, and the black-and-white movies are corresponding cross sections. For imaging details, see Materials and Methods. Scale bar: 100 μm.
(MOV)

S2 Movie Key phases of SIV development—3-D projection of mSPIM images. Related to Fig 1A–1E”. Images are projections of 3-D SPIM images extracted from a time-lapse movie, showing four key phases of SIV development at ~36, 46, 56, and 72 hpf. The stages shown correspond to models in Fig 1A–1D. The movie shows a 360° turn around the anterior-posterior axis, showing SIV plexuses on both sides of the embryo.
(AVI)

S3 Movie Outgrowth of the SIV plexus—Single cell sprouting. Related to S1A Fig. Time-lapse movie showing the first steps of the SIV plexus outgrowth in a transgenic embryo Tg(*fli1a*:EGFP)^y1^. Single endothelial cells come out of the cardinal vein and connect to form the primary SIV vessel (arrows). Images are maximal projections of 84 sections with 1.5-μm spacing. Images were acquired between ~36–54 hpf with 12 min between time points.
(AVI)

S4 Movie Outgrowth of the SIV plexus—Angiogenic sprouting. Related to S1B and S1C Fig. Time-lapse movie showing angiogenic sprouting during the SIV plexus development in a transgenic embryo Tg(*fliep*:GFF)^ubs3^;(UAS:mRFP); (*5xUAS*:*cdh5-EGFP*)^ubs12^. Sprouts grow out of the primary SIV vessel and connect to each other, forming new spots and rings of junctions. Sprout fusion happens before (first arrow) and after (second arrow) lumen opening in the SIV. Panels on the right side show the separate channels alone in black. Images are maximal projections of 85 sections with 1-μm spacing. Images were acquired between ~44–60 hpf with 15 min between time points.
(AVI)

S5 Movie Closing of a vascular loop through collateral vessel fusion in the SIV plexus. Related to S2 Fig. Time-lapse movie showing a vascular loop closing through a collateral vessel fusion in a transgenic embryo Tg(*fliep*:GFF)^ubs3^;(UAS:mRFP); (*5xUAS*:*cdh5-EGFP*)^ubs12^. A small loop made up of a major SIV branch and a small branch is resolved as the small branch approaches and fuses sidewise to the major branch. Images are maximal projections of 80 sections with 1-μm spacing. Images were acquired between 64–76 hpf with 15 min between time points.
(AVI)

S6 Movie Pruning in a developing SIV plexus—Nuclear marker. Related to Fig 2. Time-lapse movie showing pruning during SIV plexus remodeling in a double transgenic embryo TgBAC(*kdrl*:mKate2-CAAX)^ubs16^, Tg(*kdrl*:EGFPnls)^UBS1^. The endothelial cell membranes are red, and the nuclei are green. In the left panel, arrows point to pruning branches. In the right panel, key nuclei in a pruning branch are tracked over time to show cell rearrangements. Cell divisions of labeled nuclei generate new tracks. Images are maximal projections of 225 sections with 0.5-μm spacing. Images were acquired between 50–65 hpf with 15 min between time points.
(AVI)

S7 Movie Imaging of junctional markers during SIV pruning. A time-lapse movie showing pruning during SIV plexus remodeling in a transgenic embryo Tg(*fliep*:GFF)^ubs3^;(UAS:mRFP);(*5xUAS*:*cdh5-EGFP*)^ubs12.^. Cell–cell junctions are marked in green and cell cytoplasm in red. Images are maximal projections of 85 sections with 1-μm spacing. Images were acquired between ~64–82 hpf with 15 min between time points.
(AVI)

S8 Movie Junctional rearrangements in pruning type I: Lumen collapse before cell rearrangements. Related to Fig 3A. A time-lapse movie showing cell rearrangements in a pruning SIV branch in a transgenic embryo Tg(*fliep*:GFF)^ubs3^;(UAS:mRFP);(*5xUAS*:*cdh5-EGFP*)^ubs12.^. Cell–cell junctions are marked in green and cell cytoplasm in red. Black-and-white pictures show respective channels alone. Green arrows mark multicellular contacts (cell–cell junction length), and white arrows mark continuous lumen. Images are maximal projections of 80 sections with 1-μm spacing. Images were acquired between ~64–82 hpf with 15 min between time points.
(AVI)

S9 Movie Bridging cell detachment in pruning type I. Related to S3 Fig. A time-lapse movie showing final steps of cell rearrangements in pruning type I that were not captured in S8 Movie.
(AVI)

S10 Movie Junctional rearrangements in pruning type II: Cell rearrangements before lumen collapse. Related to Fig 3B. A time-lapse movie showing cell rearrangements in a pruning SIV branch in a transgenic embryo Tg(*fliep*:GFF)^ubs3^;(UAS:mRFP);(*5xUAS*:*cdh5-EGFP*)^ubs12.^. Cell–cell junctions are marked in green and cell cytoplasm in red. Black-and-white pictures show respective channels alone. Green arrows mark multicellular contacts (cell–cell junction length), and white arrows mark continuous lumen. Images are maximal projections of 80 sections with 1-μm spacing. Images were acquired between ~64–82 hpf with 15 min between time points.
(AVI)

S11 Movie Pruning in a developing SIV plexus—Cell membrane marker. Related to S4 Fig. Time-lapse movie showing pruning during SIV plexus remodeling in a transgenic embryo TgBAC(*kdrl*:mKate2-CAAX)^ubs16^. Endothelial cell membranes are black; pruning segments are pointed out with red arrows. The branches narrow down, close off the lumen, and eventually prune while detaching from the neighboring segments. Images are maximal projections of 85 sections with 1-μm spacing. Images were acquired between ~64–82 hpf with 15 min between time points.
(AVI)

S12 Movie Cell rearrangements during pruning in intersegmental vessels. Related to S5 Fig. (A) A time-lapse movie showing pruning during segmental vein formation in a transgenic embryo Tg(fliep:GFF)^ubs4^; Tg(UAS:Kaede)^rk7^. Arterial cells are marked in red, and venous cells are green (photoconverted Kaede, colors are inverted for better visualization). (B) A time-lapse movie showing pruning during segmental vein formation in a transgenic embryo Tg(*fliep*:GFF)^ubs3^;(UAS:mRFP);(UAS:EGFP-ZO-1)^ubs5^. Because of mosaic expression, only one pruning cell is labeled with EGFP-ZO-1 (green). The cytoplasm of endothelial cells is red. In both movies, the red arrow points at the pruning vessel segment, and the green arrow points at the fusing venous sprout. Images were acquired between ~36–45 hpf with 10 min between time points.
(AVI)

S13 Movie Cell self-fusion during pruning in the segmental artery. Related to Fig 4. A time-lapse movie showing cell self-fusion in a pruning intersegmental artery in a transgenic embryo Tg(*fliep*:GFF)^ubs3^;(UAS:mRFP);(UAS:EGFP-ZO-1)^ubs5^. Because of mosaic expression, only the self-fusing cell is labeled with EGFP-ZO-1 (green). The cytoplasm of endothelial cells is marked in red. The right panel shows the EGFP-ZO-1 alone (black). The movie shows formation of a unicellular tube from a flat endothelial cell (arrow). Insets false-color the apical (inner, green) and basal (outer, yellow) cell membranes at key steps before, during, and after cell self-contact formation. Images are maximal projections of 51 sections with 0.8-μm spacing. Images were acquired between ~36–51 hpf with 8 min between time points.
(AVI)

S14 Movie Lumen collapse during pruning. Related to Fig 5B. Time-lapse movie showing lumen collapse in a pruning SIV branch in a transgenic embryo TgBAC(*kdrl*:mKate2-CAAX)^ubs16^. Endothelial cell membranes are black; the lumen in the right panel is false-colored in yellow to highlight lumen instability in the pruning segment. The luminal compartments separate and reconnect multiple times before the final pruning steps occur. Images are maximal projections of 85 sections with 1-μm spacing. Images were acquired between ~64–80 hpf with 15 min between time points.
(AVI)

S15 Movie Lumen instability with high time resolution. Related to Fig 5C. Time-lapse movie showing lumen collapse in a pruning SIV branch in a transgenic embryo TgBAC(*kdrl*:mKate2-CAAX)^ubs16^. Endothelial cell membranes are black; the inner (apical/luminal) and the outer (basal) membranes are visibly separated. The upper panel shows maximal z-stack projection, and the lower panel shows a single z-slice. The luminal compartments that were initially separated reconnect, then collapse and separate, and then reconnect again. Images are maximal projections of 44 sections with 0.2-μm spacing. Images were acquired at ~66 hpf with 30 s between time points on a spinning disc microscope. Images were deconvolved using Huygens Remote Manager software.
(AVI)

S1 Table Quantification of pruning events in the SIVs of wild-type and *silent heart* morphant embryos. The table represents the quantification of all pruning events in SPIM time-lapse experiments for wild-type (A) and silent heart embryos (B). The numbers represent the three vascular loop categories defined as pruned, closed (by collateral fusion), and remaining. The graphs summarizing these results are shown in Fig 1. Minimal movie lengths were 27 h for WT and 34 h for *sih* embryos, to compensate possible developmental delay of the latter. Average values and standard deviations were calculated for both treatments. The results were analyzed using Student’s *t* test (C). See Fig 1 and S2 Movie for example time-lapse videos. See S1 Data for quantification details.
(PDF)

S2 Table Quantification of nuclei number during pruning in time-lapse experiments on transgenic embryos Tg(*kdrl*:EGFPnls)^UBS1^; Tg(BAC:*kdrl*:mKate2-CAAX)^UBS16^. Endothelial cell nuclei were quantified in regions of interest of the ISV every 10 time points starting before the first pruning event observed. The number of nuclei was increasing, corresponding to the observed cell divisions. No apoptotic nuclei were observed in the ten time-lapse experiments analyzed. See Fig 2 and S6 Movie for an example of a time-lapse video.
(PDF)

## Abbreviations

CCV: common cardinal vein
DA: dorsal aorta
dpf: days post-fertilization
GFP: green fluorescent protein
hpf: hours post-fertilization
ISV: intersegmental vessel
MDCK: Madin-Darby canine kidney
PCV: posterior cardinal vein
SeA: segmental artery
SeV: segmental vein
SIA: subintestinal artery
SIH: silent heart
SIV: subintestinal vein
SPIM: Selective Plane Illumination Microscopy
VE: vascular endothelial
WT: wild type
