# Supplementary figures and images for: Endothelial Cell Self-fusion during Vascular Pruning
Source: PLoS Biol. 2015 Apr 17;13(4):e1002126. doi: 10.1371/journal.pbio.1002126 (PMC4401649; doi:10.1371/journal.pbio.1002126)

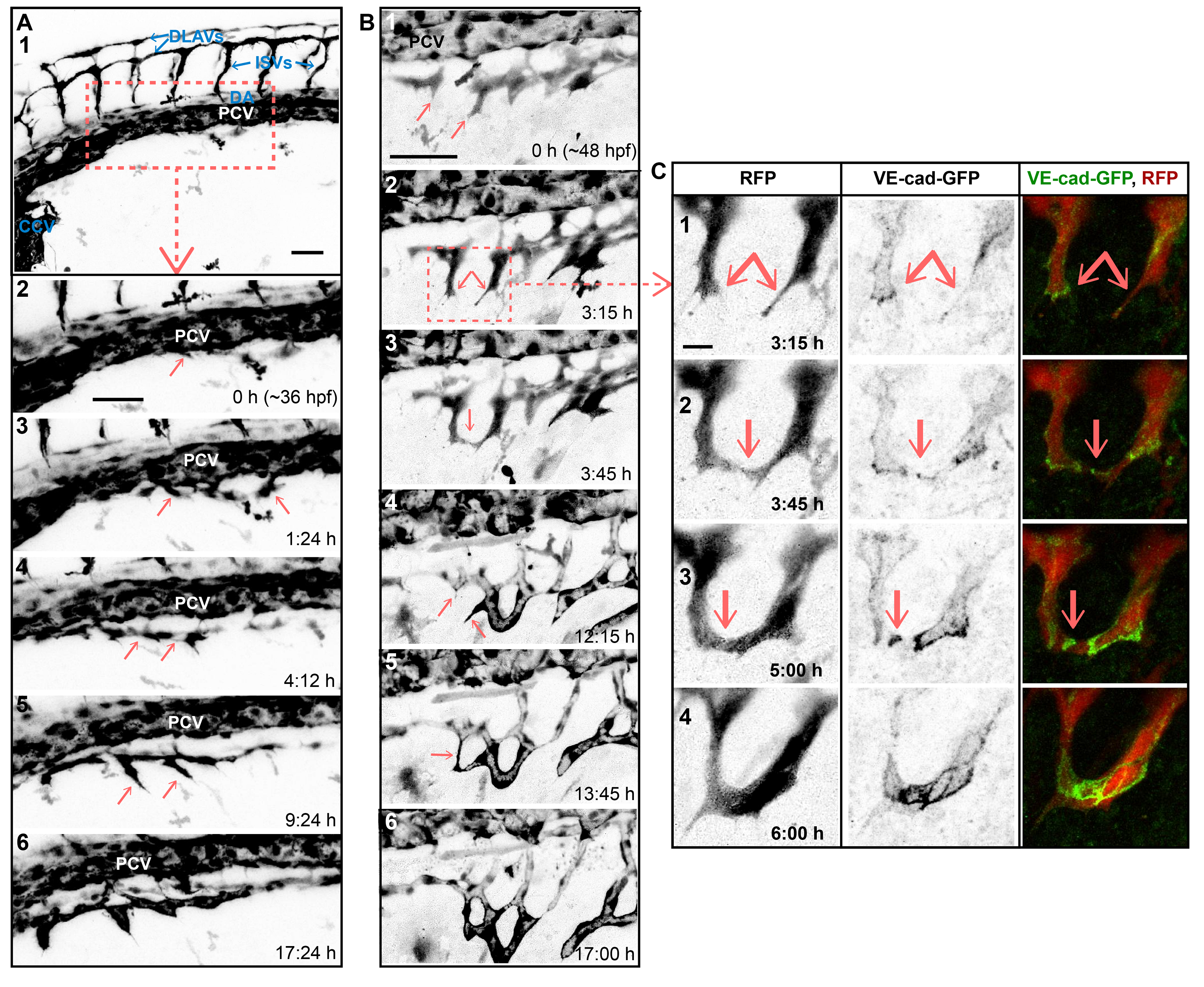

Supplement: S1 Fig — Stills of time-lapse movies showing outgrowth of the SIVs between ~36 and ~60 hpf in transgenic embryos: Tg(fli1a:EGFP) in A and Tg(fliep:GFF)ubs3,(UAS:mRFP), (5xUAS:cdh5-EGFP)ubs12 in B and C. (A) SIV emerges from the PCV at ~36 hpf (1–2). Single cells sprout ventrally from the PCV (2–3, arrows). The cells that contact each other (4, arrows) often lose contact with the PCV. A single, nonlumenized primary SIV forms and produces ventral angiogenic sprouts (5, arrows) and eventually lumenizes (6). PCV, posterior cardinal vein; DA, dorsal aorta; CCV, common cardinal vein; ISV, intersegmental vessel; DLAV, dorsal longitudinal anastomotic vessel. See also S3 Movie. (B) Multiple angiogenic sprouts originating from the primary SIV connect to each other (arrows) before (1–3) and after (4–6) lumen inflation, forming a net of vascular loops. (C) Close-up of SIV sprout fusion including the VE-cad-EGFP channel, showing new contact formation (2, arrow marks a spot of junctions), contact expansion (3, a ring of junctions), and transformation of the new branch into a multicellular tube (lines of junctions in 4). See also S4 Movie. Scale bars: 50 μm (A and B) and 10 μm (C). (TIF) [file pbio.1002126.s002.tif]

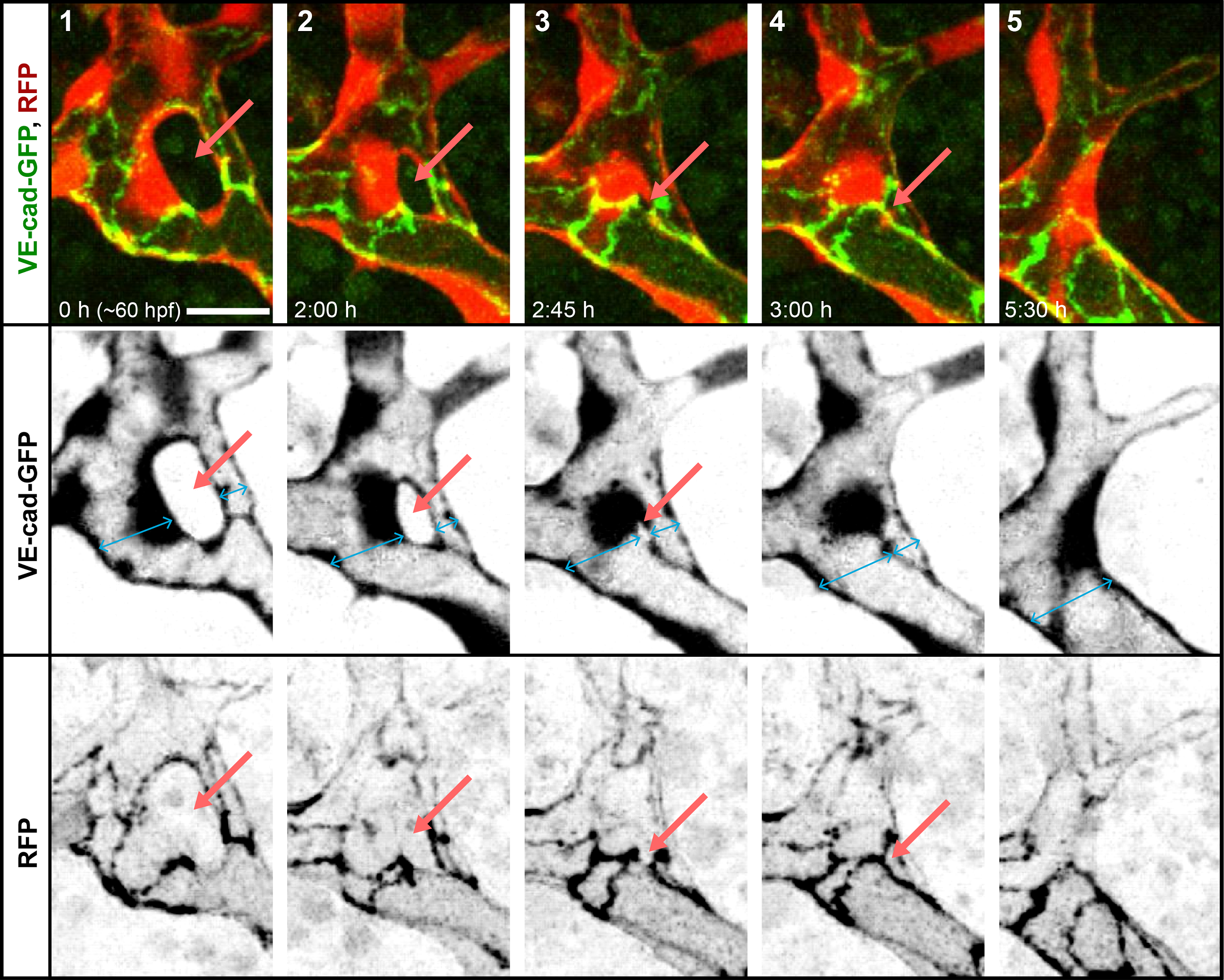

Supplement: S2 Fig — A small branch forms a vascular loop with the major branches (1, arrow). The opening of the loop shrinks as the small branch approaches the major branch sidewise (2–3, arrows). The last very small opening is closed as the side walls of two vessels touch (5) and eventually connect to form a single lumen (6). Fusing lumens of the branches are marked with blue arrows. Scale bars: 10 μm. See also S5 Movie. (TIF) [file pbio.1002126.s003.tif]

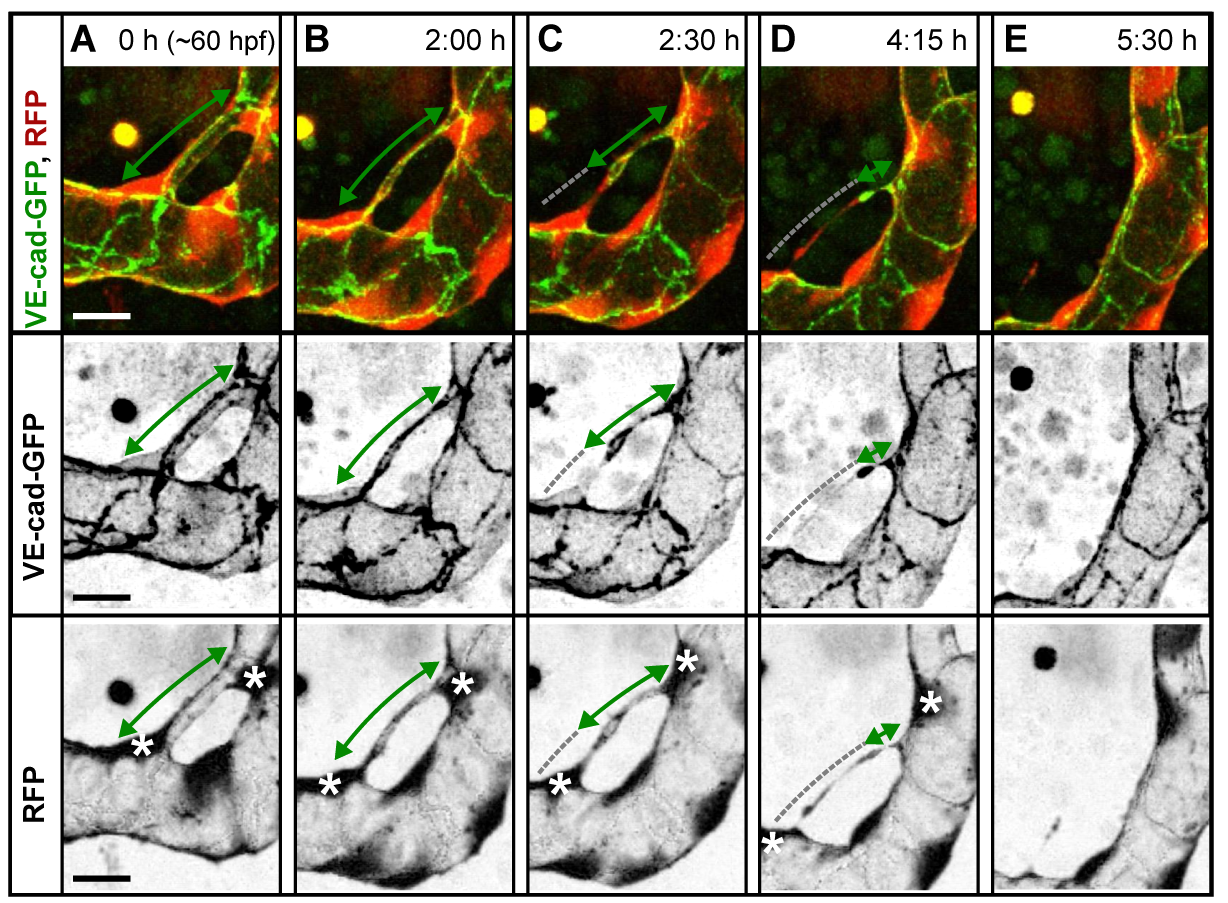

Supplement: S3 Fig — A small, lumenized branch is made of two cells (A). Lumen collapses when the branch is still multicellular (B); after lumen collapse, cells move away from each other, and the cell–cell contact surface shrinks (C). The last cell–cell contact (D, green arrow) is eventually resolved completely as the last cytoplasmic extention of the bridging cell detaches from the major branch (E). See also S9 Movie. (TIF) [file pbio.1002126.s004.tif]

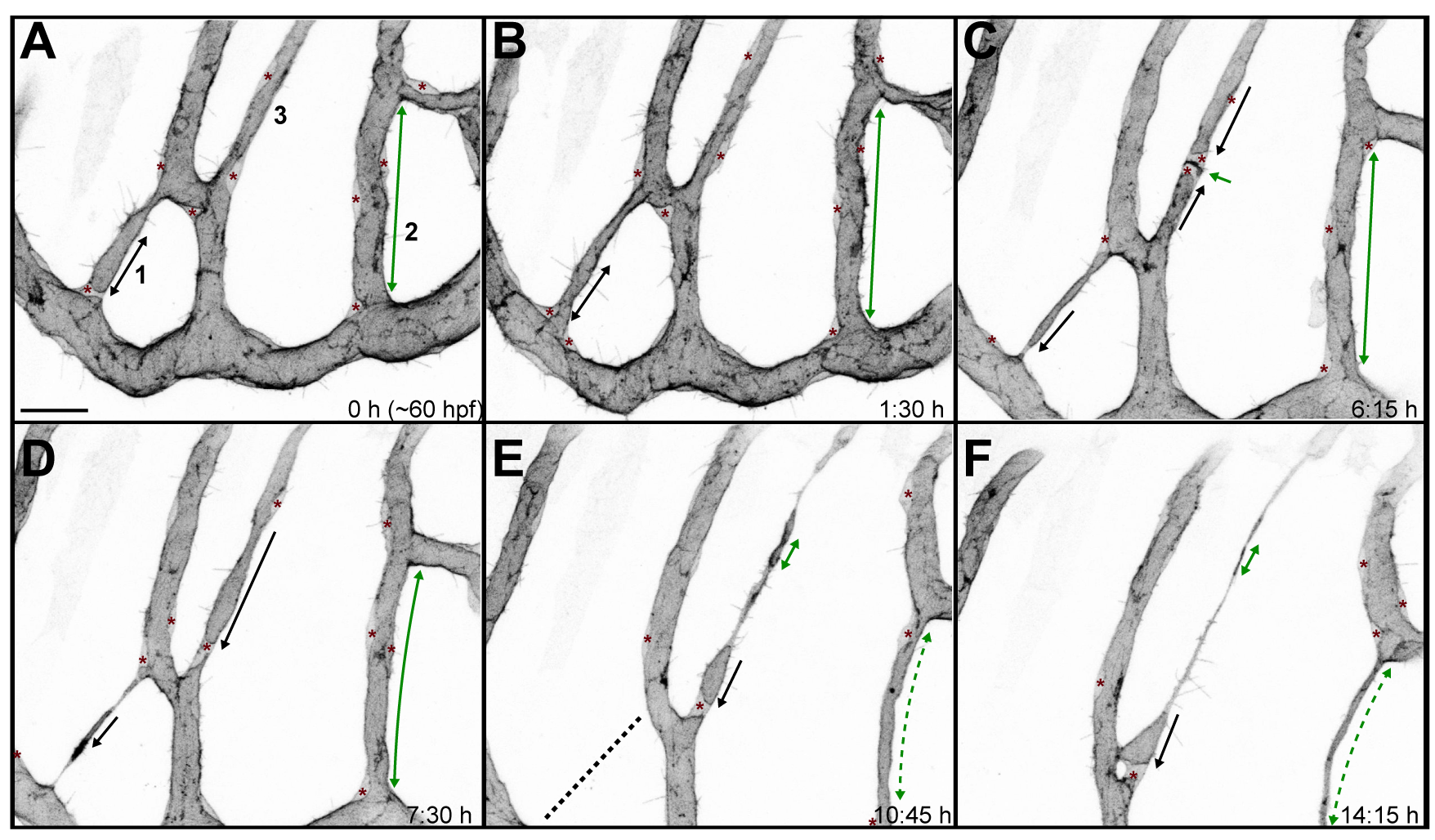

Supplement: S4 Fig — The TgBAC(kdrl:mKate-CAAX) transgenic line allows visualization of the endothelial cell membranes. Transcellular lumen formation and collapse can be distinguished by the presence of inflated, round-ended, apical membrane compartments (black arrows). In certain cases, it is also possible to recognize multicellular vessel fragments by the increase in staining density at the cell–cell contact surfaces (green arrows). The nuclei are recognizable as round structures that are brighter because the two membranes (apical and basal) are well separated around the nucleus (red asterisks in the vessels of interest). These criteria were used for quantifications presented in Fig 3C. Three pruning vessel segments are followed in this figure. Segment 1: lumen in a unicellular vessel fragment (black arrow) is parted next to the nucleus (red asterisk). The lumen perfuses (B) and deflates again (C) to finally collapse completely (D) when the vessel detaches (E, dotted line). Segment 2: a multicellular vessel fragment with the cell–cell contacts visible as darker lines of the membrane staining (A–D, green arrows) narrows significantly when the lumen collapses (E–F). Cells leave the branch after the lumen collapse (D–F, asterisks mark the nuclei). Segment 3: a unicellular vessel fragment undergoes cell division generating two nuclei (C, asterisks), a new cell–cell contact surface (C, green arrow), and two luminal compartments (C, black arrows). The lumen is partially restored (D) but later collapses, leaving a small vacuole-like structure (E, black arrow). The last cell–cell contact surface is reduced as the pruning proceeds (E–F). See also S11 Movie. (TIF) [file pbio.1002126.s005.tif]

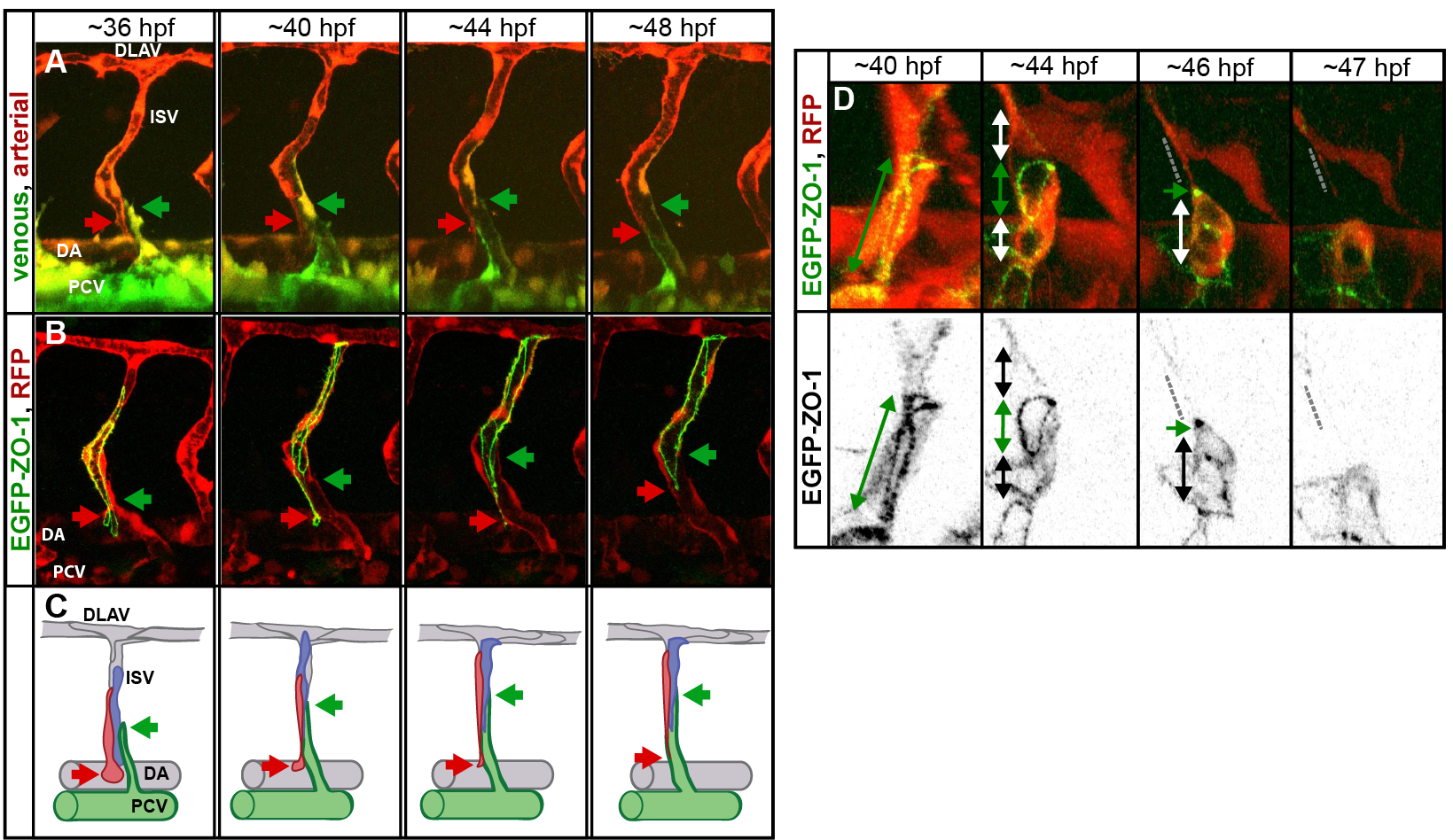

Supplement: S5 Fig — (A) Stills of a time-lapse movie showing segmental vein formation in a transgenic embryo Tg(fliep:GFF)ubs4; Tg(UAS:Kaede)rk7. Arterial cells are marked in red, venous cells are green (photoconverted Kaede, colors are inverted for better visualization). A new venous sprout (green arrow) grows out of the PCV towards a segmental vessel (red arrow). The venous sprout connects to the ISV. At the same time, the ISV segment connected to the aorta narrows and eventually detaches as the cells migrate up to contribute to the dorsal part of the ISV. See also S12 Movie. (B) Still images of the segmental vein formation in a transgenic embryo Tg(fliep:GFF)ubs4; Tg(UAS:mRFP);Tg(UAS:EGFP-ZO-1)ubs5. A single cell is expressing the EGFP-ZO-1 (green). The cell belongs to the ISV and is anchored in the DA (red arrow points to the ring-like junction). When the venous sprout connects (green arrow), the green cell moves up the ISV. The junctional ring gradually reduces in size to a spot, and the cell eventually detaches from the aorta and migrates up the ISV. See also S13 Movie. (C) A cellular model of pruning during the segmental vein formation. Arteries are grey, and venous cells are green. Red and blue cells are initially connected to the DA. As the venous sprout attaches to the ISV, the arterial cells detach from the DA and move up to contribute to the dorsal part of the ISV. (D) Stills from a time-lapse movie showing multicellular-to-unicellular tube transformation during pruning in an ISV in a transgenic embryo Tg(fliep:GFF)ubs4; Tg(UAS:mRFP);Tg(UAS:EGFP-ZO-1)ubs5. Key steps of pruning are shown, corresponding to the model in Fig 6. Green arrows mark multicellular contacts (cell–cell junction length), white arrows mark transcellular lumen, and grey dotted lines mark unicellular fragments without lumen. (TIF) [file pbio.1002126.s006.tif]

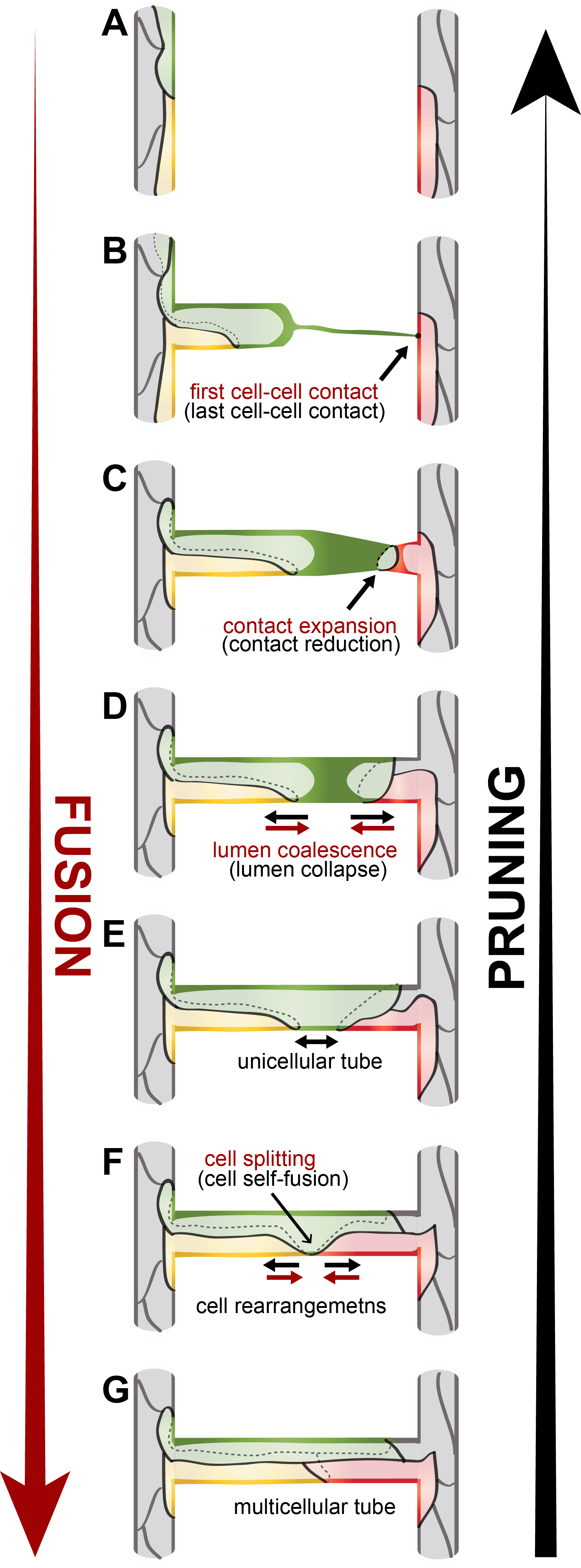

Supplement: S6 Fig — Cell rearrangements and shape and polarity changes happen in reversed order during vessel pruning (black writing), as compared to vessel fusion (red writing). (A–C) Formation of a new contact involves de novo apical polarization and expansion of a new junctional connection. A corresponding last pruning step involves shrinking and resolution of the junctional contact, which also implicates removal of the apical membrane compartment. (D) Expansion of apical membrane compartments leads to lumen coalescence and opening. The inversed process leads to lumen collapse and separation of a continuous apical compartment into two separate ones. (E) A unicellular tube is a transient structure that forms through invagination and fusion of the apical membrane (during vessel fusion) or through cell self-fusion (during pruning). (F–G) Cell self-fusion is accompanied by migration of neighboring cells away to opposite sides. Splitting of a unicellular tube, resembling a “reversed” self-fusion, is accompanied by neighboring cell migration towards the cell. (TIF) [file pbio.1002126.s007.tif]
