# Supplementary material for: Endothelial Cell Self-fusion during Vascular Pruning
Source: PLoS Biol. 2015 Apr 17;13(4):e1002126. doi: 10.1371/journal.pbio.1002126 (PMC4401649; doi:10.1371/journal.pbio.1002126)
Supplement: S1 Table — The table represents the quantification of all pruning events in SPIM time-lapse experiments for wild-type (A) and silent heart embryos (B). The numbers represent the three vascular loop categories defined as pruned, closed (by collateral fusion), and remaining. The graphs summarizing these results are shown in Fig 1. Minimal movie lengths were 27 h for WT and 34 h for sih embryos, to compensate possible developmental delay of the latter. Average values and standard deviations were calculated for both treatments. The results were analyzed using Student’s t test (C). See Fig 1 and S2 Movie for example time-lapse videos. See S1 Data for quantification details. (PDF) [file pbio.1002126.s023.pdf]

A	Movie No.	Prune d	Closed	Remainin g	Sum	Nr of time points	Movie duration (h)
	WT 1	3	0	0	3	328	54.67
	WT 2	1	1	1	3	281	46.83
	WT 3	1	0	0	1	281	46.83
	WT 4	5	1	0	6	261	43.5
	WT 5	1	1	1	3	260	43.33
	WT 6	3	1	1	5	260	43.33
	WT 7	4	1	0	5	260	43.33
	WT 8	3	0	0	3	259	43.17
	WT 9	3	1	0	4	241	40.17
	WT 10	2	0	0	2	241	40.17
	WT 11	3	2	1	6	241	40.17
	WT 12	1	1	1	3	231	38.5
	WT 13	2	2	1	5	188	31.33
	WT 14	4	0	1	5	188	31.33
	WT 15	3	2	0	5	178	29.67
	WT 16	2	0	1	3	178	29.67
	WT 17	0	1	1	2	178	29.67
	WT 18	3	0	0	3	163	27.17
	WT 19	6	0	1	7	162	27
	Average	2.63	0.74	0.53	3.89		
	SD	1.46	0.71	0.50	1.55		

B	Movie No.	Prune d	Close d	Remainin g	Sum	Nr of time points	Movie duration (h)
	SIH 1	0	1	2	3	263	43.83
	SIH 2	1	2	2	5	249	41.5
	SIH 3	0	2	2	4	249	41.5
	SIH 4	1	1	3	5	234	39
	SIH 5	1	2	2	5	225	37.5
	SIH 6	0	1	2	3	225	37.5
	SIH 7	0	0	2	2	225	37.5
	SIH 8	1	4	3	8	208	34.67
	SIH 9	0	0	1	1	206	34.33
	Average	0.44	1.44	2.11	4.00		
	SD	0.50	1.17	0.57	1.94		

C	Compared populations	T-Student test value
	Pruned WT to pruned SIH	0.000007
	Remaining WT to remaining SIH	0.000009
	Closed WT to closed SIH	0.141
