# Supplementary material for: Endothelial Cell Self-fusion during Vascular Pruning
Source: PLoS Biol. 2015 Apr 17;13(4):e1002126. doi: 10.1371/journal.pbio.1002126 (PMC4401649; doi:10.1371/journal.pbio.1002126)
Supplement: S2 Table — Endothelial cell nuclei were quantified in regions of interest of the ISV every 10 time points starting before the first pruning event observed. The number of nuclei was increasing, corresponding to the observed cell divisions. No apoptotic nuclei were observed in the ten time-lapse experiments analyzed. See Fig 2 and S6 Movie for an example of a time-lapse video. (PDF) [file pbio.1002126.s024.pdf]

Movie No.	Number of nuclei in the ROI (Relative time points)						Nr of pruning events
	T=0	+10 tp	+20 tp	+30 tp	+40 tp	+50 tp	
**1**	28	28	31	32			2
**2**	10	12	14	14			2
**3**	26	28	31	32	34	34	2
**4**	18	20	23	23	23		1
**5**	14	16	17	17			3
**6**	18	22	25	25			1
**7**	18	18	20	20			2
**8**	8	10	11	12			2
**9**	20	20	21	22			2
**10**	14	16	18	18			2
